# Microbial Diversity in Sediment Ecosystems (Evaporites Domes, Microbial Mats, and Crusts) of Hypersaline Laguna Tebenquiche, Salar de Atacama, Chile

**DOI:** 10.3389/fmicb.2016.01284

**Published:** 2016-08-22

**Authors:** Ana B. Fernandez, Maria C. Rasuk, Pieter T. Visscher, Manuel Contreras, Fernando Novoa, Daniel G. Poire, Molly M. Patterson, Antonio Ventosa, Maria E. Farias

**Affiliations:** ^1^Laboratorio de Investigaciones Microbiológicas de Lagunas Andinas, Planta Piloto de Procesos Industriales Microbiológicos, Centro Científico Tecnológico, CONICETTucumán, Argentina; ^2^Department of Marine Sciences, University of ConnecticutGroton, CT, USA; ^3^Australian Centre for Astrobiology, University of New South WalesSydney, NSW, Australia; ^4^Centro de Ecología AplicadaSantiago, Chile; ^5^Centro de Investigaciones Geológicas, Universidad Nacional de La Plata-ConicetLa Plata, Argentina; ^6^Department of Microbiology and Parasitology, Faculty of Pharmacy, University of SevillaSevilla, Spain

**Keywords:** hypersaline lakes, microbial mats, endoevaporites, concretions, Atacama, pyrosequencing

## Abstract

We combined nucleic acid-based molecular methods, biogeochemical measurements, and physicochemical characteristics to investigate microbial sedimentary ecosystems of Laguna Tebenquiche, Atacama Desert, Chile. Molecular diversity, and biogeochemistry of hypersaline microbial mats, rhizome-associated concretions, and an endoevaporite were compared with: The V4 hypervariable region of the 16S rRNA gene was amplified by pyrosequencing to analyze the total microbial diversity (i.e., bacteria and archaea) in bulk samples, and in addition, in detail on a millimeter scale in one microbial mat and in one evaporite. Archaea were more abundant than bacteria. *Euryarchaeota* was one of the most abundant phyla in all samples, and particularly dominant (97% of total diversity) in the most lithified ecosystem, the evaporite. Most of the euryarchaeal OTUs could be assigned to the class *Halobacteria* or anaerobic and methanogenic archaea. *Planctomycetes* potentially also play a key role in mats and rhizome-associated concretions, notably the aerobic organoheterotroph members of the class *Phycisphaerae*. In addition to cyanobacteria, members of *Chromatiales* and possibly the candidate family *Chlorotrichaceae* contributed to photosynthetic carbon fixation. Other abundant uncultured taxa such as the candidate division MSBL1, the uncultured MBGB, and the phylum *Acetothermia* potentially play an important metabolic role in these ecosystems. Lithifying microbial mats contained calcium carbonate precipitates, whereas endoevoporites consisted of gypsum, and halite. Biogeochemical measurements revealed that based on depth profiles of O_2_ and sulfide, metabolic activities were much higher in the non-lithifying mat (peaking in the least lithified systems) than in lithifying mats with the lowest activity in endoevaporites. This trend in decreasing microbial activity reflects the increase in salinity, which may play an important role in the biodiversity.

## Introduction

The Salar de Atacama, located in the Chilean Central Andes, is the largest Quaternary halite deposit in the world (3064 km^2^ and >900 m thick; Warren, 2010). This Salar is comprised of a porous halide (90%), the interstices of which are permeated with a sodium chloride brine rich in lithium, potassium, magnesium, and boron (Bevacqua, 1992; Risacher and Alonso, 1996). Laguna Tebenquiche, located in the northern part close to the core zone of the Salar (Risacher et al., 2003), is one of the largest water bodies in this system (Demergasso et al., 2008). The lake is fed by groundwater of Tertiary and Quaternary volcanic origin (Risacher and Alonso, 1996). Bacterial and archaeal microorganisms inhabiting this lake are subject to extreme environmental conditions, such as high solar radiation (incl. UV), extreme diel temperature fluctuations, extreme changes in salinity due to net evaporation, and high lithium, boron, and arsenic concentrations (Lara et al., 2012; Farías et al., 2014). “Extreme” environmental conditions are generally conducive of microbial mat development (Rothschild and Mancinelli, 2001; Dupraz and Visscher, 2005) and a variety of photosynthetic organosedimentary ecosystems were found in Laguna Tebenquiche (Farías et al., 2014). These ecosystems included non-lithifying laminated photosynthetic microbial mats characterized by copious amounts of exopolymeric substances similar to other hypersaline mats (Dupraz et al., 2004, 2009) and also endoevaporitic domes made up of gypsum harboring endolithic phototrophs such as those described by Canfield et al. (2004) and Oren et al. (1995).

Previous studies based on culture-dependent methods recovered a large number of bacterial and archaeal strains from Laguna Tebenquiche's water column and sediments. Isolates included moderately halophilic Gram-negative rods (Prado et al., 1991), moderately halophilic Gram-positive cocci (Valderrama et al., 1991), heterotrophic halophilic microorganisms (Prado et al., 1993), and extremely halophilic archaea (Lizama et al., 2001, 2002). Using morphological and physiological properties, the bacterial isolates were assigned to the genera *Vibrio, Halomonas, Acinetobacter, Alteromonas, Psychrobacter*, and *Marinococcus*, all of which grouped within the class *Gammaproteobacteria*. Archaeal strains were assigned to the genera *Halorubrum, Haloarcula, Halobacterium*, and *Haloferax* (phylum *Euryarchaeota*).

Demergasso et al. (2008) analyzed the bacterial community of the water column by DGGE fingerprinting at several locations of Laguna Tebenquiche during the winter and summer season. Their study revealed a heterogeneous community composition of which changed along a salinity gradient in the water column of the lake. The dominant phylum was *Bacteroidetes*, which in the most saline part of the lake comprised a cluster related to *Salinibacter* relatives and at intermediate salinities consisted of clusters distantly related to *Psychroflexus* spp. Within the *Gammaproteobacteria*, which encompassed the most abundant class, a cluster related to uncultured bacteria from Mono Lake (USA) dominated. In addition, a few clones could be assigned to the candidate division OP1 (currently reclassified within the phylum *Parcubacteria*), to uncultivated clones CS_B020 and BD1-5 from marine sediments and to sequences of the KB1 group found in sediments from hypersaline brines.

Recently, we reported on the bacterial diversity, mineral composition, and key metabolic activities in two lakes in the Salar de Atacama (Farías et al., 2014), Laguna La Brava, and Laguna Tebenquiche. In both lakes, discrete bacterial communities, and mineral compositions developed along the salinity gradient of the overlying water. In Laguna Tebenquiche, the most abundant bacterial 16S rRNA amplicons in mats and endoevaporites resembled *Bacteroidetes*, and the second-most abundant amplicons could be assigned to *Proteobacteria (Alphaproteobacteria and Deltaproteobacteria)*. Surprisingly low cyanobacterial diversity was found, which was corroborated by a low abundance of chlorophyll a (Chl*a*). Another recent study, focusing on phototrophic bacteria (Thiel et al., 2010) was motivated by the low Chl*a* concentration and low abundance of cyanobacteria in Laguna Tebenquiche. That investigation found evidence for a new gammaproteobacterial lineage based on *pufLM* gene analyses and furthermore found that green sulfur bacteria could not be detected with molecular techniques but could be revealed by culture-dependent methods.

Our previous study focused on the bacterial 16S rRNA sequences in two lakes and included a non-lithifying microbial mat and a gypsum endoevaporite in Laguna Tebenquiche (Farías et al., 2014). In a consecutive field campaign documented here, and with the purpose of covering all microbial ecosystems associated to mineral precipitation, we increased the number different benthic microbial ecosystems to include one endoevaporitic domal mat, two microbial mats with different degrees of lithification, and two rhizome-associated concretions. These concretions were included in our study because they present lithified structures similar to microbialites, but in contrast form in association with plants. Also, in the present investigation we deployed primers, which amplified the V4 hypervariable region of both archaeal and bacterial 16S rDNA genes, in order to cover the total microbial diversity in each system with the purpose to investigate the low cyanobacterial presence determined in a previous study, as well as the vertical distribution of key functional groups, we determined the microbial diversity in discrete depth horizons in a non-lithifying microbial mat and an endoevaporitic mat. Our investigation enables a correlation of taxonomic diversity with geochemical gradients (e.g., oxygen and sulfide profiles) on a small (vertical) scale and with physicochemical and geochemical characteristics (e.g., salinity, water depth) on a large (horizontal) scale. The results of this study increase our knowledge of the genetic and metabolic diversity of the benthic microbial ecosystems in Laguna Tebenquiche and provide novel insights into the microbial processes in extreme ecosystems at high altitude.

## Materials and methods

### Sample collection

**Figure 1:**
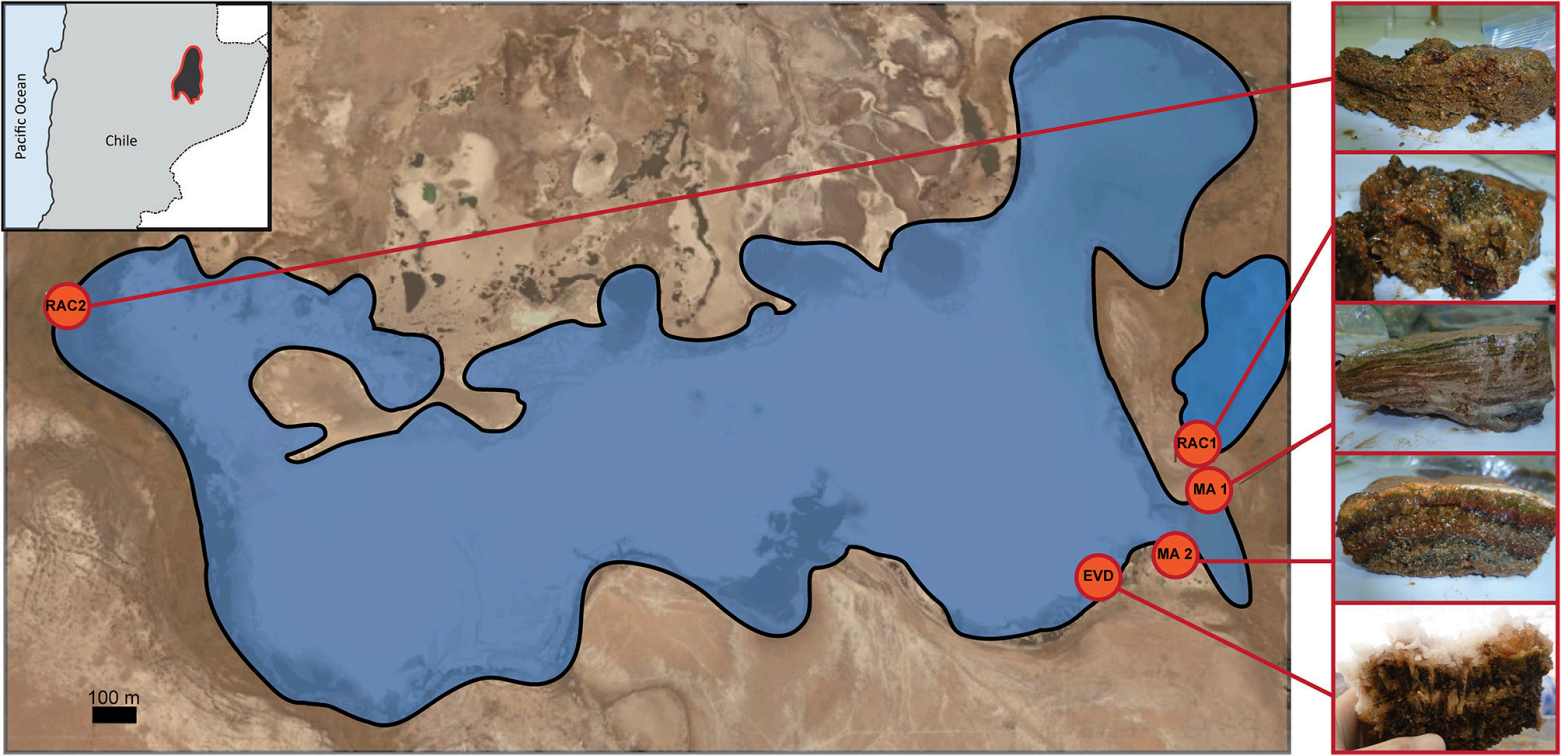
**Location of microbial mats (MA1 and MA2), rhizome-associated lithified concretions (RAC1 and RAC2), and evaporite (EVD) in Laguna Tebenquiche**.

Samples were obtained from Laguna Tebenquiche in November 2013 and selected based on a preliminary inspection of sedimentary structures present along a salinity gradient (Figure 1). Five different locations were sampled: one endoevaporitic dome, EVD (23°08′24.6″ S, 68°15′0.2″ W); two microbial mats, MA1 (23°08′18.5″S, 68°14′49.9″W) and MA2 (23°08′23.44″S, 68°14′53.89″W); and two rhizome-associated concretions, RAC1 (23°08′15.42″S, 68°14′49.89″W) and RAC2 (23°7′47.50″S, 68°16′22.8″W) from opposite sides of the lake. Water samples (1 L) were collected immediately (ca. 1 cm above) over the sampling sites of the corresponding sediment systems. The endoevaporitic gypsum dome was recovered from ca. 15 cm water depth. The organic-rich microbial mats MA1 and MA2 were submersed in ca. 15 and 5 cm of water, respectively. MA1 was very gelatinous and contained only a few trapped minerals, contrary to MA2, which was leathery, and comprised a semi-lithified subsurface layer. The rhizome-associated concretions forming around the root system of the grass *Distichlis spicata* are commonly found near the shoreline of the lake. Samples for a detailed depth analysis of the microbial diversity were collected from the MA1 and EVD sites, respectively, which represent the end members of lithification (non-lithified and fully lithified, respectively).

### Water column characteristics

The temperature and pH of the water column were determined *in situ*. Samples were stored in acid-cleaned bottles on ice in the dark until analyses in the laboratory within 48 h. Dissolved oxygen, salinity, conductivity, total P, ${\text{NO}}_{3}^{-}$, ${\text{NO}}_{2}^{-}$, dissolved Si, Ca^2+^, Mg^2+^, K^+^, ${\text{SO}}_{4}^{2-}$, and Na^+^, according to the methodology described by Eaton et al. (2005). ${\text{NH}}_{4}^{+}$, orthophosphates, and Total Organic Nitrogen (TON) were analyzed using a Merck Nova 60 Spectro Photometer by following standard methods, as described by American Public Health Association (1998).

### Sediment characteristics

Bulk samples of all microbial sediments were taken for mineral analyses and kept at 4°C in the dark prior to analysis. The mineral composition was determined by X-ray diffraction (XRD) analysis of finely ground (<20 μm) samples of dried mats and endoevaporites with a PANalyticalX'Pert PRO diffractometer, with Cu lamp (kα = 1.5403 Å) operated at 40 mÅ, and 40 kV at Centro de Investigaciones Geológicas (La Plata, Argentina).

### Microelectrode measurements

Depth profiles of the oxygen and sulfide concentration were measured *in situ* (Taillefert and Rozan, 2002; Visscher et al., 2002) during the peak of photosynthesis when the intensity of photosynthetically active radiation (PAR) was 1850–2550 μmol quanta.m^−2^.s^−1^. Oxygen was determined with a Clark-type probe and sulfide using an amperometric sensor (Unisense, Arhus, Denmark). Both O_2_ and H_2_S needle probes had internal reference, guard, and measuring electrodes and were connected to a modified portable picoammeter (Unisense PA 2000, Arhus, Denmark). The electrodes were calibrated in the laboratory before and after field measurements and in between measurements checked by a two-point calibration. Electrodes could not be deployed in rhizome-associated lithified concretions due to the hardness of these samples. Three to five replicate profiles covering the upper 10–15 mm of each sample were determined. The oxygen and sulfide concentrations were corrected for altitude according to Sherwood et al. (1991).

### DNA extraction and sequencing

For DNA analyses, triplicate cores (2 cm^2^ each) were taken to a depth of 3 cm and pooled prior to homogenizing in order to obtain a representative sample. For the depth distribution of diversity, samples of MA1, and EVD were dissected following visible layers with depth: MA1 layers were taken from 0 to 1.5 mm (layer 1), 1.5 to 4 mm (layer 2), 4 to 6.5 mm (layer 3), 6.5 to 9.5 mm (layer 4), and 9.5 to 11.5 mm (layer 5). EVD samples were taken from 0 to 10 mm (layer 1; predominantly the halite crust), 10 to 20 mm (layer 2; the gypsum crust); 20 to 30 mm (layer 3; top of the endolithic mat), 30 to 37 mm (layer 4; bottom of the endolithic mat), 37 to 57 mm (layer 5; sediment underlying the mat). Homogenates used for DNA extraction were stored at −20°C in the dark and processed within a week.

Total DNA was isolated from 0.2 g material following the protocol supplied in the Power Biofilm DNA Isolation Kit (MO BIO Laboratories, Inc.).

The V4 hypervariable region of the bacterial and archaeal 16S rRNA gene was amplified using the RK primers (F515 and R806) that contain adaptors A and B required for 454 FLX pyrosequencing (Roche Applied Science) and a 10 nucleotide “multiple identifier” (MID; Bates et al., 2010). Bates et al. (2010) designed this primer set to be universal for a broad range of archaeal and bacterial taxa with few biases or excluded groups. Five independent PCRs were performed to reduce bias. The PCR mixture (25 μl final volume) contained 2.5 μl FastStart High Fidelity 10X Reaction Buffer (Roche Applied Science, Mannheim, Germany), 20 ng of template DNA, 0.4 μM of each primer, and 1.25 U FastStart High Fidelity Enzyme Blend (Roche Applied Science), and 0.2 mM dNTPs. The PCR conditions were 95°C for 5 min for initial denaturalization, followed by 95°C for 45 s, 57°C for 45 s, 72°C for 60 s in 30 cycles, and a final elongation step at 72°C for 4 min. Two negative control reactions containing all components except the template were performed. The five reactions products were pooled and purified using AMPure beads XP. Quantification of the purified PCR product was performed using the Quant-IT Pico Green dsDNA Kit (Invitrogen Molecular Probes Inc, Oregon, USA). Purified PCR product was pyrosequenced on a Roche 454 GS-FLX system, Titanium chemistry. Sequence data have been deposited in the NCBI Sequence Read Archive (SRA) under the accession number: SRP066553.

### 16S rRNA amplicons processing

All analyses of the V4 hypervariable region of the microbial 16S rRNA amplicons were conducted within the QIIME software package (Caporaso et al., 2010). Raw 454 reads were demultiplexed and quality filtered by removing low quality or ambiguous reads. Sequences shorter than 150 bp were discarded and Roche adapters, linkers, primers, and sample barcodes were removed. The 454 reads were denoised to reduce possible sequencing errors and clustered at 97% identity in operational taxonomic unit (OTU) using uclust (Edgar, 2010). One representative sequence of each cluster was aligned to the Greengenes database with PyNAST 1.1 (DeSantis et al., 2006). Chimeric sequences were detected using the ChimeraSlayer algorithm and subsequently removed (Haas et al., 2011). OTUs observed in only one sample or represented by only one sequence were discarded. Finally, the number of sequences assigned to each OTU was summarized in a table generated by QIIME.

In order to analyze the diversity, the OTU table was subsampled using 10 replicates for each sampling effort at increasing intervals of 100 sequences. Alpha diversity indexes were calculated on each subsample and on the OTU table. Alpha diversity metrics calculated included Observed OTUs, Chao1 (estimates the species richness), Shannon (the entropic information of the abundances of observed OTUs, accounting for both richness and evenness), Equitability (Shannon index corrected for the number of species, “pure” evenness), Dominance (calculated as the sum of the squares of the frequencies of each OTU), and Simpson (1-Dominance) indexes.

### Canonical correspondence analysis

A constrained ordination was carried out by a Canonical Correspondence Analysis (CCA) to correlate environmental variables with microbial phyla and samples. A Monte Carlo test with 499 permutations was carried out to ensure the significance of canonical axes. CANOCO 4.5 software package (Microcomputer Power, Ithaca, NY, USA) was used to perform the CCA and the tool CANODRAW for triplot visualization (ter Braak and Smilauer, 2002).

## Results

### Environmental characteristics

#### Water column

Physicochemical analyses of the water column overlying the sediment samples (Table 1; Table S1; no data were obtained for RAC2) showed an increase of the salinity (i.e., conductivity) from the RAC1 site to the MA2 site, with intermediate values for sites MA1 and EVD. This observation was supported by trends in total alkalinity as well as major cation (e.g., sodium, magnesium) and anion (e.g., chloride, sulfate) concentrations. The calcium concentration was the highest at the RAC1 site, intermediate at the MA1, and EVD sites and the lowest at the MA2 site, which pattern followed the amount of calcium incorporation in minerals present in these samples (i.e., no calcium was incorporated in MA2). The phosphate concentration was the highest in RAC1, intermediate in MA1 and EVD, and the lowest in MA2 water samples. Nitrate concentrations followed the opposite trend, with the highest values in water overlying MA2 and the lowest values present at RAC1. Metals and metalloids such as lithium, and boron where in lower amount in RAC1 where conductivity is lower, arsenic is almost constant in all the samples sites while silica is lower only in MA2 (Table S1).

**Table 1:** **Physico-chemical parameters for the overlying water from the different samples studied**.

**Physico-chemical parameter**	**Unit**	**MA1**	**MA2**	**RAC1**	**EVD**
Biochemical oxygen demand (BOD)	mg/L	3.9	1.9	4.0	4.2
Chemical oxygen demand (COD)	mg/L	269	191	169	224
Chlorophyll a	μg/L	<0.1	<0.1	<0.1	2
Conductivity	mS/cm	161	228	94	177
Hardness	mg/L	14,909	31,785	7640	16,662
pH	–	7.8	7.6	7.4	7.8
Total Alkalinity	mg CaCO_3_/L	487	697	380	525
Temperature	°C	23.3	27.0	27.6	31.0
Turbidity	NTU	15.17	4.40	4.52	19.11
Salinity	g/L	106	150	62	117
Organic matter	mg/L	17	14	7	12

#### Sedimentary structures

**Figure 2:**
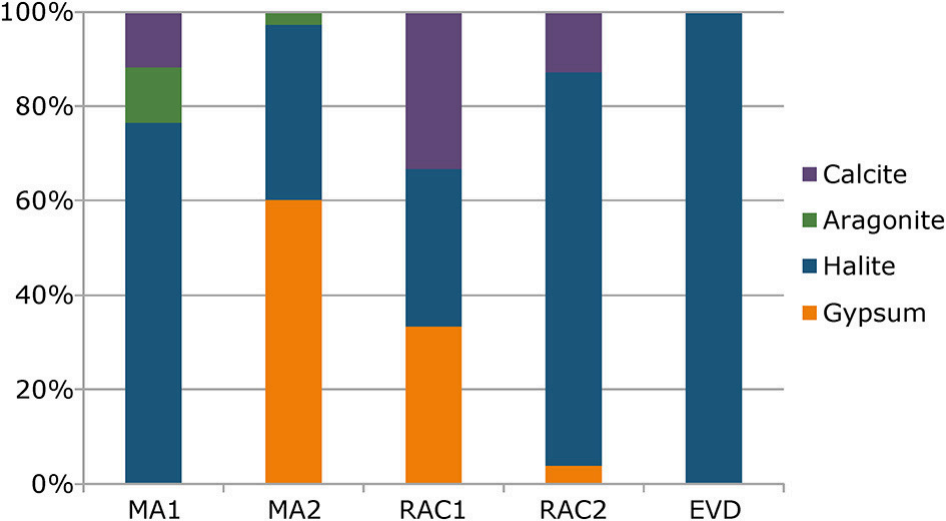
**Mineral composition of microbial mats (MA1 and MA2), rhizome-associated lithified concretions (RAC1 and RAC2), and evaporite (EVD) obtained by X-ray diffraction (XRD) analyses**.

In the microbial sedimentary structures, the total mineral content per volume (corresponding to the degree of lithification) was the highest in EVD and RAC1, intermediate in MA2 and the lowest in the non-lithifying MA1 samples. The XRD analysis of mineralogy revealed that RAC1 and RAC2 comprised halite and to a lesser extent gypsum and calcite (Figure 2). MA2 contained gypsum and halite with traces of aragonite and calcite, MA1 mainly halite with lower amounts of calcite, and aragonite. EVD consisted of predominantly of gypsum, with halite being less abundant. It should be noted that a fraction of minerals, especially halite, observed in MA1 and MA2 could be due to drying artifacts. Hand samples, inspected under a dissection microscope showed calcite grains, as demonstrated by dissolution upon addition of 2N HCl, but with the exception of EVD did not reveal halite as a major mineral component. We postulate that the hypersaline porewater upon drying of the samples, cause halite to precipitate.

**Figure 3:**
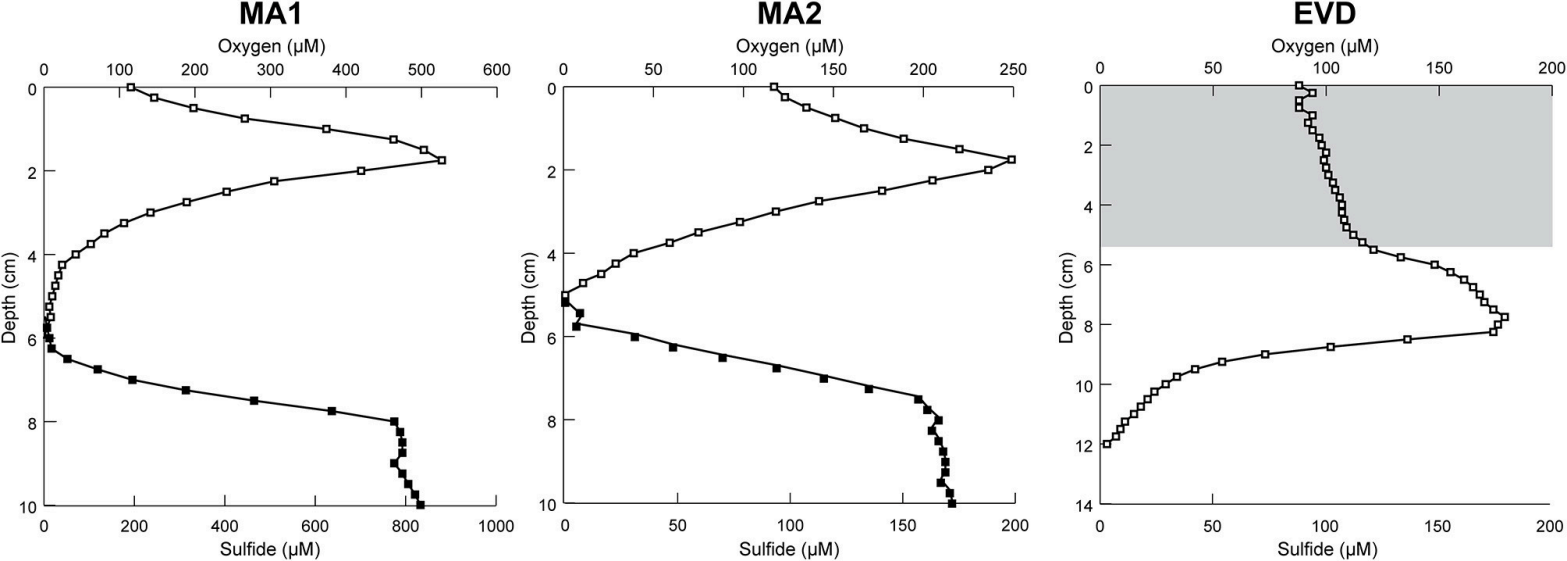
**Representative depth profiles of the concentration of O_**2**_ (***squares***) and sulfide (***triangles***) measured ***in situ*** with microelectrodes during the light period**. The gray shaded area depicts the mineral crust in RAC1.

Oxygen and sulfide concentrations were measured in MA1, MA2, and EVD (Figure 3). The oxygen concentration in both mats peaked at 1.75–2 mm depth (527 and 249 μM in MA1 and MA2, respectively) and decreased to zero at 5.25–6 mm. The higher O_2_ peak in MA1 compared to MA2 was indicative of a higher rate of photosynthesis, and the steepness of the O_2_ depth profile suggested high respiration and/or sulfide oxidation rates. Much higher sulfide concentrations were observed in MA1 compared to MA2 (830 and 173 μM, respectively), supporting the notion of higher metabolic activities in MA1. A 4–8 mm thick salt crust (gray shaded area) covered the surface of EVD, and a metabolically-active endolithic mat was present underneath this halite layer. The oxygen concentration increased to 179 μM at 7.75 mm, after which it slowly decrease to zero at ca. 12 mm. No sulfide could be detected in the upper 15 mm of EVD.

### Composition of bacterial and archaeal communities

A total of 19,320 raw reads were obtained from all five samples by using 454 pyrosequencing. After quality filtering, denoising, and removing chimeras, a total of 15,438 rRNA sequences representing 926 OTUs clustered at 97% sequence similarity remained. The rarefaction curves of observed OTUs vs. sequence number per sample are shown in Figure S1. Rarefaction curves revealed that only the EVD sample approached saturation, indicating that the sequencing depths for the other samples were insufficient to cover the microbial diversity. Using the rarefied or random subsampled sequences (i.e., 1070 per sample to remove sampling depth heterogeneity), the highest and the lowest number of OTUs were observed in RAC1 and EVD, respectively (Table S2). The diversity of microbial communities in the samples was further evaluated using Chao1, Shannon, Equitability, Dominance, and Simpson indexes. The Shannon, Equitability, and Simpson indexes ranged from 3.00 to 6.55, 0.814 to 0.456 and 0.963 to 0.595, respectively and showed the highest values in RAC1 and the lowest values in EVD. However, of all samples studied the Chao index was the highest in MA1 and RAC1. This implies that the samples RAC1 and MA1 have the highest microbial diversity with a larger equitability of OTUs and the sample EVD has the lowest microbial diversity with a few dominant OTUs.

**Figure 4:**
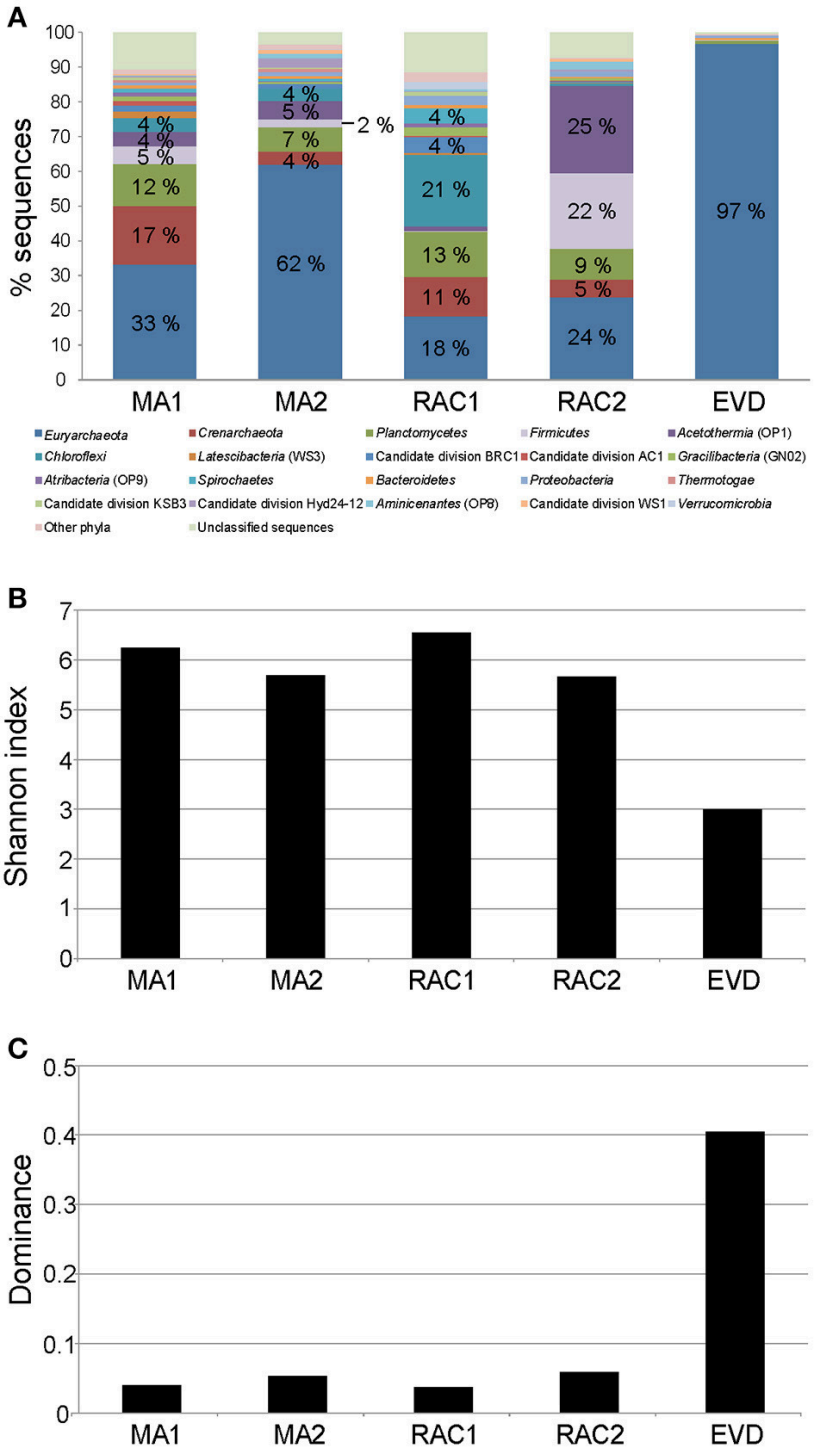
**Comparison of the microbial diversity in microbial mats (MA1 and MA2), rhizome-associated lithified concretions (RAC1 and RAC2) and endoevaporite (EVD). (A)** Stacked column graph representing the relative distribution of the dominant phyla in the different samples. Sequences were assigned taxonomically using Greengenes database with a minimum percentage similarity of 97% and a minimum *e*-value of 10^−5^. **(B)** Shannon index of the estimated richness of OTUs. **(C)** Dominance index of OTUs.

Most of the 16S rRNA sequences in EVD were classified as archaea (97%). Furthermore, EVD was the only sample in which all sequences could be assigned to a domain level. All samples were composed of several phyla or groups, however, only a few were dominant (Figure 4). The *Euryarchaeota* phylum was ubiquitous in all samples, notably EVD where it comprised 97% of 16S rRNA sequences. This high proportion of euryarchaeal sequences was also reflected in a low diversity and in a large dominance of a few OTUs in EVD. Both mat samples MA1 and MA2 showed a relatively similar phylum composition, differing only in the respective proportions, with *Euryarchaeota* being the most abundant (33 and 62% of 16S rRNA sequences in MA1 and MA2, respectively). Other phyla present in these samples include *Crenarchaeota, Planctomycetes, Firmicutes, Acetothermia*, and *Chloroflexi*. The rhizome-associated concretions RAC1 and RAC2 exhibited disparate 16S rRNA profiles. The most abundant phylum in RAC1 was *Chloroflexi* (21% of 16S rRNA sequences) and in the two dominant phyla in RAC2 were *Acetothermia* and *Firmicutes* (accounting for 25 and 22% of 16S rRNA sequences, respectively). *Euryarchaeota, Crenarchaeota*, and *Planctomycetes* were present in both rhizome-associated concretions but comprised lower percentages.

OTUs related to the phylum *Cyanobacteria*, well known for their important role in mats (Visscher et al., 1991, 1992; van Gemerden, 1993; Baumgartner et al., 2009b), were detected in low number in all samples.

The most abundant OTUs (higher than 1% 16S rRNA sequences) were assigned to taxonomic levels (Table 2). In MA1, the most abundant phylum, *Euryarchaeota*, was made up by five OTUs, three of which belonging to the class *Thermoplasmata*, within which one OTU classified as DHVEG-1 containing 23% of the 16S rRNA sequences, and two OTUs to the class *Methanobacteria*. Within *Crenarchaeota*, one OTU was assigned to the marine benthic group B (MBGB) with 16% of the 16S rRNA sequences. Two of three abundant OTUs from the phylum *Planctomycetes* were related to the class *Phycisphaerae*, and two of the *Firmicutes* OTUs belonged to the family *Halobacteroidaceae*, with one of these specifically classified to the genus *Halanaerobacter* (1% 16S rRNA sequences). The taxa *Acetothermia, Chloroflexi* (class *Anaerolineae*), *Latescibacteria*, candidate division AC1, *Gracilibacteria*, and *Atribacteria* were represented in MA1 by one OTU with more than 1% of the total number of 16S rRNA sequences.

**Table 2:** **Abundant microbial OTUs per sample classified at the lowest possible taxonomic level**.

**MA1**		**MA2**		**RAC1**		**RAC2**		**EVD**	
DHVEG-1 (Phyl. *Euryarchaeota*/ Class *Thermoplasmata*)	23.1	DHVEG-1 (Phyl. *Euryarchaeota*/ Class *Thermoplasmata*)	19.8	Cand. fam. *Chlorothrixaceae* (Phyl. *Chloroflexi*/ Class *Chloroflexia*)	17.1	KB1 (Phyl. *Acetothermia*)	25.0	Gen. *Halonotius* (Phyl. *Euryarchaeota*/ Class *Halobacteria*)	62.7
MBGB (Phyl. *Crenarchaeota*)	16.4	Gen. *Halonotius* (Phyl. *Euryarchaeota*/ Class *Halobacteria*)	14.8	DHVEG-1 (Phyl. *Euryarchaeota*/ Class *Thermoplasmata*)	12.6	Gen. *Halanaerobium* (Phyl. *Firmicutes*/ Class *Clostridia*)	16.2	Gen. *Halorhabdus* (Phyl. *Euryarchaeota*/ Class *Halobacteria*)	6.4
AKAU3564 (Phyl. *Planctomycetes*/ Class *Phycisphaerae*)	6.9	Fam. *Halobacteriaceae* (Phyl. *Euryarchaeota*/ Class *Halobacteria*)	6.5	MBGB (Phyl. *Crenarchaeota*)	10.0	AKAU3564 (Phyl. *Planctomycetes*/ Class *Phycisphaerae*)	6.5	Fam. *Halobacteriaceae* (Phyl. *Euryarchaeota*/ Class *Halobacteria*)	6.2
KB1 (Phyl. *Acetothermia*)	4.1	KB1 (Phyl. *Acetothermia*)	5.3	AKAU3564 (Phyl. *Planctomycetes*/ Class *Phycisphaerae*)	4.5	MBGB (Phyl. *Crenarchaeota*)	5.2	Gen. *Halorubrum* (Phyl. *Euryarchaeota*/ Class *Halobacteria*)	5.4
Fam. *Halanaerobiaceae* (Phyl. *Firmicutes*/ Class *Clostridia*)	3.2	Gen. *Halorhabdus* (Phyl. *Euryarchaeota*/ Class *Halobacteria*)	4.3	Uncultured soil bacterium PRR-11 (Cand. div. BRC1)	2.1	Fam. *Halanaerobiaceae* (Phyl. *Firmicutes*/ Class *Clostridia*)	3.8	XKL75 (Phyl. *Euryarchaeota*/ Class *Halobacteria*)	5.2
20c–4 (Phyl. *Euryarchaeota*/ Class *Thermoplasmata*)	2.8	Gen. *Halorubrum* (Phyl. *Euryarchaeota*/ Class *Halobacteria*)	4.2	NPL-UPA2 (Cand. div. BRC1)	2.0	Fam. *Halobacteriaceae* (Phyl. *Euryarchaeota*/ Class *Halobacteria*)	3.7	Gen. *Haloarcula* (Phyl. *Euryarchaeota*/ Class *Halobacteria*)	4.0
OPB11 (Phyl. *Chloroflexi*/ Class *Anaerolineae*)	2.6	XKL75 (Phyl. *Euryarchaeota*/ Class *Halobacteria*)	4.1	Fam. *Methanomassiliicoccaceae* (Phyl. *Euryarchaeota*/ Class *Thermoplasmata*)	1.9	Cand. div. MSBL1 (Phyl. *Euryarchaeota*)	3.6	MSP41 (Phyl. *Euryarchaeota*/ Ord. *Halobacteriales*)	2.4
MSBL9 (Phyl. *Planctomycetes*/ Class *Phycisphaerae*)	2.2	MBGB (Phyl. *Crenarchaeota*)	3.7	3BR-5F (Phyl. *Gracilibacteria*)	1.8	Fam. *Methanomassiliicoccaceae* (Phyl. *Euryarchaeota*/ Class *Thermoplasmata*)	3.5	Gen. *Haloplanus* (Phyl. *Euryarchaeota*/ Class *Halobacteria*)	1.3
Ord. *Methanobacteriales* (Phyl. *Euryarchaeota*/ Class *Methanobacteria*)	1.8	Cand. fam. *Chlorothrixaceae* (Phyl. *Chloroflexi*/ Class *Chloroflexia*)	2.9	MSBL9 (Phyl. *Planctomycetes*/ Class *Phycisphaerae*)	1.6	Gen. *Halonotius* (Phyl. *Euryarchaeota*/ Class *Halobacteria*)	2.5		
Uncultured soil bacterium PRR-12 (Phyl. *Latescibacteria*)	1.7	AKAU3564 (Phyl. *Planctomycetes*/ Class *Phycisphaerae*)	2.3	Fam *Pirellulaceae* (Phyl. *Planctomycetes*/ Class *Planctomycetia*)	1.5	Gen. *Halorhabdus* (Phyl. *Euryarchaeota*/ Class *Halobacteria*)	2.4		
Fam. *Methanomassiliicoccaceae* (Phyl. *Euryarchaeota*/ Class *Thermoplasmata*)	1.5	WM88 (Cand. div. Hyd24-12)	2.3	SBYZ_6080 (Phyl. *Spirochaetes*)	1.5	ArcA07 (Phyl. *Euryarchaeota*)	2.3		
B04R032 (Cand. div. AC1)	1.5	Fam. *Halobacteriaceae* (Phyl. *Euryarchaeota*/ Class *Halobacteria*)	2.0	Ord. *Phycisphaerales* (Phyl. *Planctomycetes*/ Class *Phycisphaerae*)	1.3	HMMVPog-54 (Phyl. *Aminicenantes*)	2.0		
Gen. *Halanaerobacter* (Phyl. *Firmicutes*/ Class *Clostridia*)	1.4	MSP41 (Phyl. *Euryarchaeota*/ Ord. *Halobacteriales*)	1.5	Ord. *Methanobacteriales* (Phyl. *Euryarchaeota*/ Class *Methanobacteria*)	1.2	Gen. *Halanaerobacter* (Phyl. *Firmicutes*/ Class *Clostridia*)	1.3		
3BR–5F (Phyl. *Gracilibacteria*)	1.2	HMMVPog-54 (Phyl. *Aminicenantes*)	1.4	BA021 (*Atribacteria*)	1.1	XKL75 (Phyl. *Euryarchaeota*/ Fam. *Halobacteriaceae*)	1.1		
BA021 (Phyl. *Atribacteria*)	1.2	Gen. *Haloarcula* (Phyl. *Euryarchaeota*/ Class *Halobacteria*)	1.4	Sediment-4 (Phyl. *Spirochaetes*/ Ord. *Leptospirales*)	1.1				
Phyl. *Planctomycetes*	1.1	Cand. div. WS1	1.2	Uncultured crenarchaeote MCG (Phyl. *Crenarchaeota*)	1.1				
Cand. div. MSBL1 (Phyl. *Euryarchaeota*)	1.1	Phyl. *Planctomycetes*	1.1	MSBL6 (Phyl. *Acetothermia*)	1.0				
		SC103 (Phyl. *Thermotogae*/ Fam. *Thermotogaceae*)	1.0	ODP1230B3009 (Phyl. *Planctomycetes*/ Class *Phycisphaerae*)	1.0				

*Each OTU contains at less 1% 16S rRNA sequences*.

In MA2, nine OTUs could be assigned to *Euryarchaeota*, one of which with 19% 16S rRNA sequences to the class *Thermoplasmata*. The remaining OTUs were assigned to the orders *Halobacteriales* and *Haloferacales* with four specifically to the genera *Halorhabdus* and *Haloarcula* and *Halonotius* and *Halorubrum*, respectively (4 and 1 and 15 and 4% 16S rRNA sequences, respectively). One OTU in MA2 was related to the crenarchaeotal MBGB (4% 16S rRNA sequences), two OTUs were related to *Planctomycetes*, one of which to the class *Phycisphaerae* (2% 16S rRNA sequences), and *Acetothermia, Chloroflexi* (candidate family *Chlorothrixaceae*), candidate division Hyd24-12, *Aminicenantes*, candidate division WS1, and *Thermotogae* were represented by one OTU.

In RAC1, the dominant phylum *Chloroflexi* was represented by one OTU, which accounted for 17% of the 16S rRNA sequences. Three abundant euryarchaeal OTUs were found in this sample, two OTUs were assigned to *Thermoplasmata* and one OTU to the class *Methanobacteria*. Four of five OTUs classified into *Planctomycetes* were assigned to the class *Phycisphaerae* and the remaining OTU to the class *Planctomycetia* (family *Pirellulaceae*). Other OTUs were assigned to candidate division BRC1 (two OTUs), *Gracilibacteria* (one OTU), *Spirochaetes* (two OTUs), and *Acetothermia* (one OTU). RAC2 comprised one *Acetothermia* OTU (with 25% 16S rRNA sequences), seven euryarchaeotal OTUs related to the classes *Halobacteria* (two of these OTUs were assigned to the genera *Halorhabdus* and *Halonotius*), *Methanobacteria* and *Thermoplasmata*; and three *Firmicutes* OTUs classified to the family *Halanaerobiaceae* (two of which to the genera *Halanaerobium* and *Halanaerobacter*). One OTU each with more than 1% of the total 16S rRNA sequences was assigned to the phyla *Planctomycetes, Crenarchaeota* and *Aminicenantes*, respectively.

In the EVD sample, all dominant OTUs belonged to the orders *Halobacteriales* and *Haloferacales*, some of which could be classified to the genera *Halorhabdus* and *Haloarcula* and *Halonotius, Halorubrum*, and *Haloplanus*, respectively (6 and 4 and 63, 5, and 1% 16S rRNA sequences, respectively).

The OTUs related to the taxa MBGB (*Crenarchaeota*), AKAU3564 (phylum *Planctomycetes*, class *Phycisphaerae*) and KB1 or MSBL6 (*Acetothermia*) were present in mat and rhizome-associated concretion samples. A dominance of DHVEG-1 (phylum *Euryarchaeota*, class *Thermoplasmata*) was observed in the mat samples and RAC1.

### Microbial structure and their relation to water physicochemical parameters

**Figure 5:**
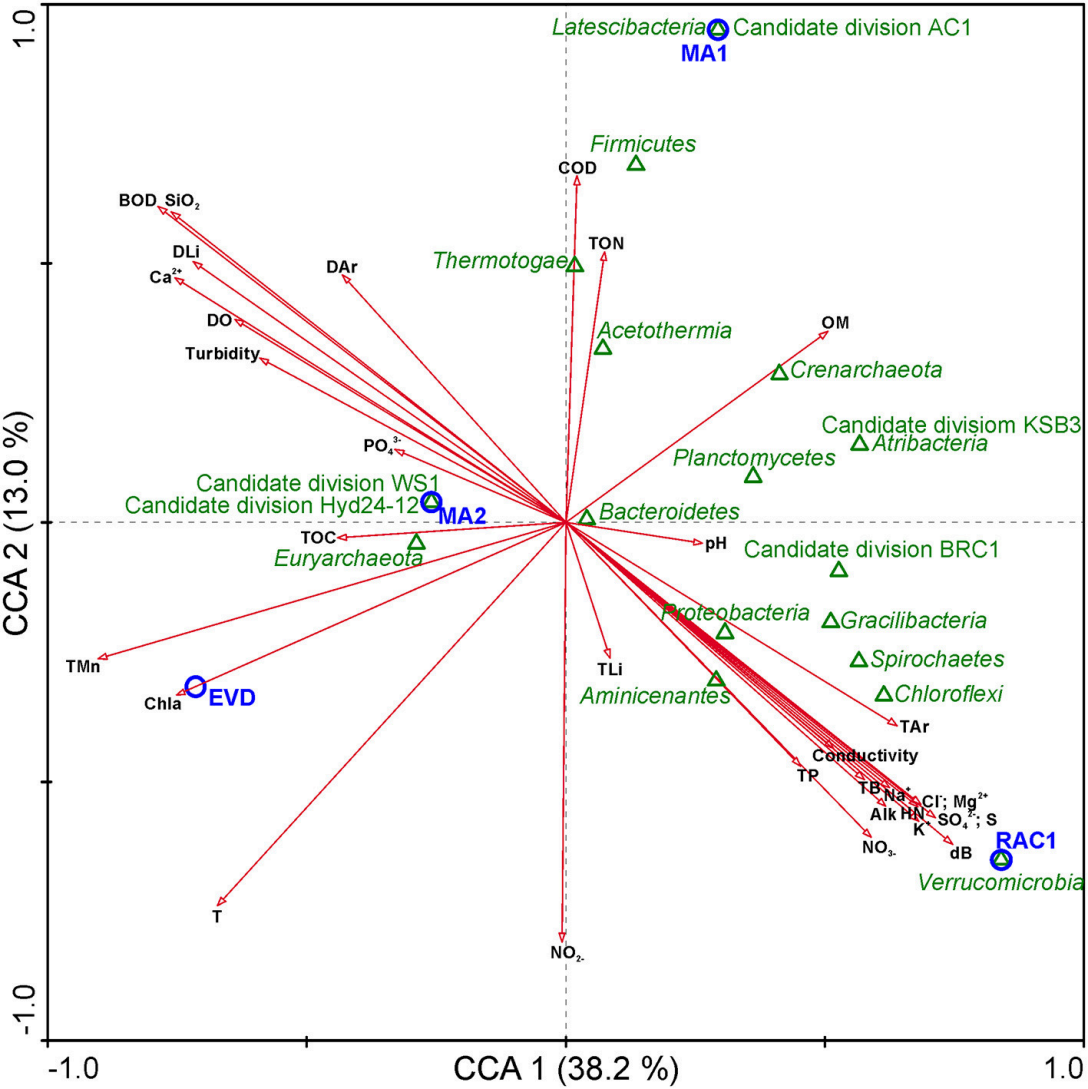
**Canonical correspondence analysis (CCA) of microbial community, samples, and environmental parameters**. Arrows indicate the direction and magnitude of environmental parameters associated with phyla (open green triangles) and samples studied (open blue circles). BOD, Biochemical oxygen demand; COD, Chemical oxygen demand; Chl*a*, Chlorophyll a; HN, Hardness; Alk, Total alkalinity; T, Temperature; OM, Organic matter; TOC, Total organic Carbon; DO, Dissolved oxygen; ${\text{NO}}_{3}^{-}$, Nitrate; ${\text{NO}}_{2}^{-}$, Nitrite; TON, Total organic nitrogen; TP, Total phosphorus; OP, Orthophosphate; ${\text{SO}}_{4}^{2-}$, Sulfate; S, Sulfur; S^2−^, Total sulfide; Na^+^, Sodium; Cl^−^, Chloride; K^+^, Potassium; Mg^2+^, Magnesium; Ca^2+^, Calcium; DB, Dissolved boron; TB, Total boron; DLi, Dissolved lithium; TLi, Total lithium; SiO_2_, Silica; Dar, Dissolved Arsenic; Tar, Total Arsenic.

A canonical correspondence analysis (CCA) was carried out to investigate the relationships between several physicochemical and geochemical characteristics and the 10 most abundant phyla present in each different sample (Figure 5). In this analysis, CCA1, and CCA2 ordination axes could explain 51.2% of the total variance data. Conductivity is positively correlated to geochemical properties such as sodium, chloride, potassium, magnesium, sulfate, and nitrate concentrations and the hardness of the water appeared stronger correlated to magnesium than to calcium. Such physicochemical characteristics were negatively correlated with RAC1 and with representatives of the phylum *Verrucomicrobia* in this sample. The relative proportion of sequences assigned to *Euryarchaeota*, in each sample, which peaked in samples EVD and MA2, could likely be contributed to an increase in conductivity. Biodiversity in MA2 showed a negative correlation with TON but a positive correlation with the nitrite and nitrate concentration. *Bacteroidetes* was a minor phylum in all samples and in the CCA triplot this phylum appeared in the center, suggesting that this phylum prevailed under a wide range of physicochemical conditions.

### Phylogenetic distribution with depth

The mat sample MA1 and the endoevaporite sample EVD were selected for a more detailed study as representatives of ecosystems in opposite conditions lithification. The samples were dissected according to the uppermost five visible layers. We obtained a total of 35,383 raw reads from the two samples combined (i.e., 10 layers). A total of 27,479 rRNA sequences that passed sequence processing (i.e., quality control criteria, denoising, and removing chimeras) were clustered in 1018 OTUs with a minimum of 97% sequence similarity. The rarefaction curves for MA1 and EVD samples with depth revealed that the surface layer and layer second to the surface of both samples reached an asymptote. In contrast, the bottom three layers in both samples did not reach an asymptote, suggesting that the sequencing depths in these samples were insufficient (Figure S2). The number of sequences was normalized to 1900 sequences per layer in order to allow comparison of the different depth horizons in MA1 and EVD (Table 3). In both samples, the lowest number of OTUs was detected in the surface layer (layer 1). The largest number of OTUs was found in the middle (third) layer of MA1 and in the bottom layer (layer 5) of EVD. The Shannon diversity index and Chao1 estimator confirmed that in MA1 the lowest microbial diversity with a high dominance was located in the top layer. The highest diversity was found in the third layer. The oxic-anoxic interface during the daytime fell within this layer and likely facultative and thus obligate anaerobic and microaerophilic microorganisms proliferated here. In EVD, Shannon, Chao1, and Simpson indices all increased with depth. The surface layer in both samples exhibited the lowest diversity with a few strongly dominant OTUs, likely resulting from the most extreme physicochemical conditions to which this layer was exposed.

**Table 3:** **Observed microbial richness and diversity estimates based on 97% OTU clusters by layers in MA1 and EVD**.

**Sample**	**Layer**	**Number of total reads**	**Seqs/Sample**	**Observed OTUs**	**Chao1**	**Shannon**	**Equitability**	**Dominance**	**Simpson**
MA1	1	2340	1900	121	154	4.437	0.641	0.154	0.846
	2	2685	1900	159	183	5.284	0.723	0.065	0.935
	3	2348	1900	316	443	6.721	0.809	0.025	0.975
	4	3668	1900	177	280	5.176	0.693	0.067	0.933
	5	2091	1900	283	408	6.217	0.763	0.042	0.958
EVD	1	3259	1900	77	102	2.927	0.467	0.317	0.683
	2	2625	1900	106	138	3.294	0.490	0.331	0.669
	3	3196	1900	197	239	5.624	0.738	0.071	0.929
	4	1903	1900	193	246	5.861	0.772	0.049	0.951
	5	3364	1900	241	301	5.606	0.709	0.082	0.918

**Figure 6:**
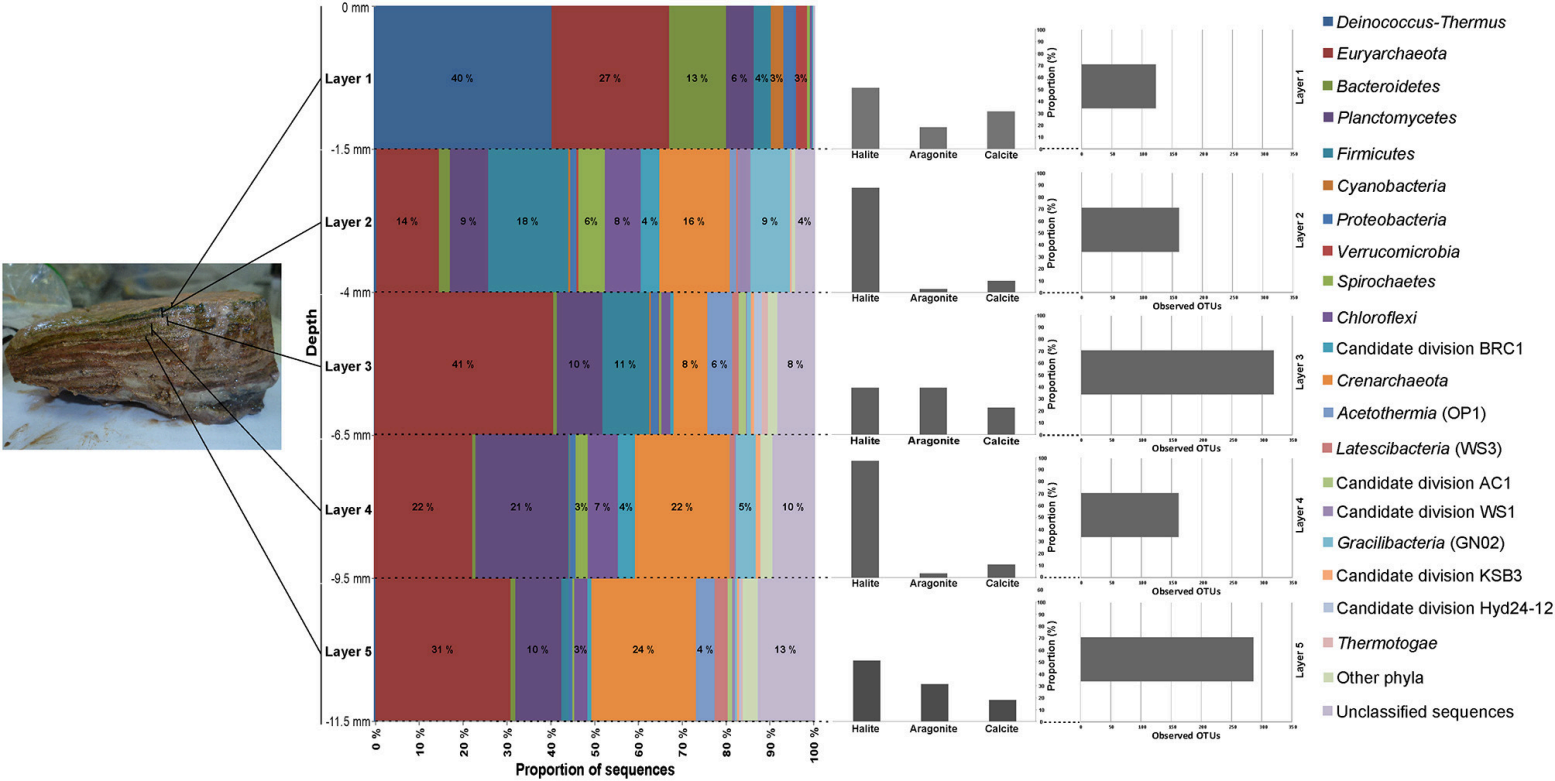
**Taxonomic composition by layers in MA1**. Sequences were assigned taxonomically using Greengenes database with a minimum percentage similarity of 97% and a minimum *e*-value of 10^−5^.

**Figure 7:**
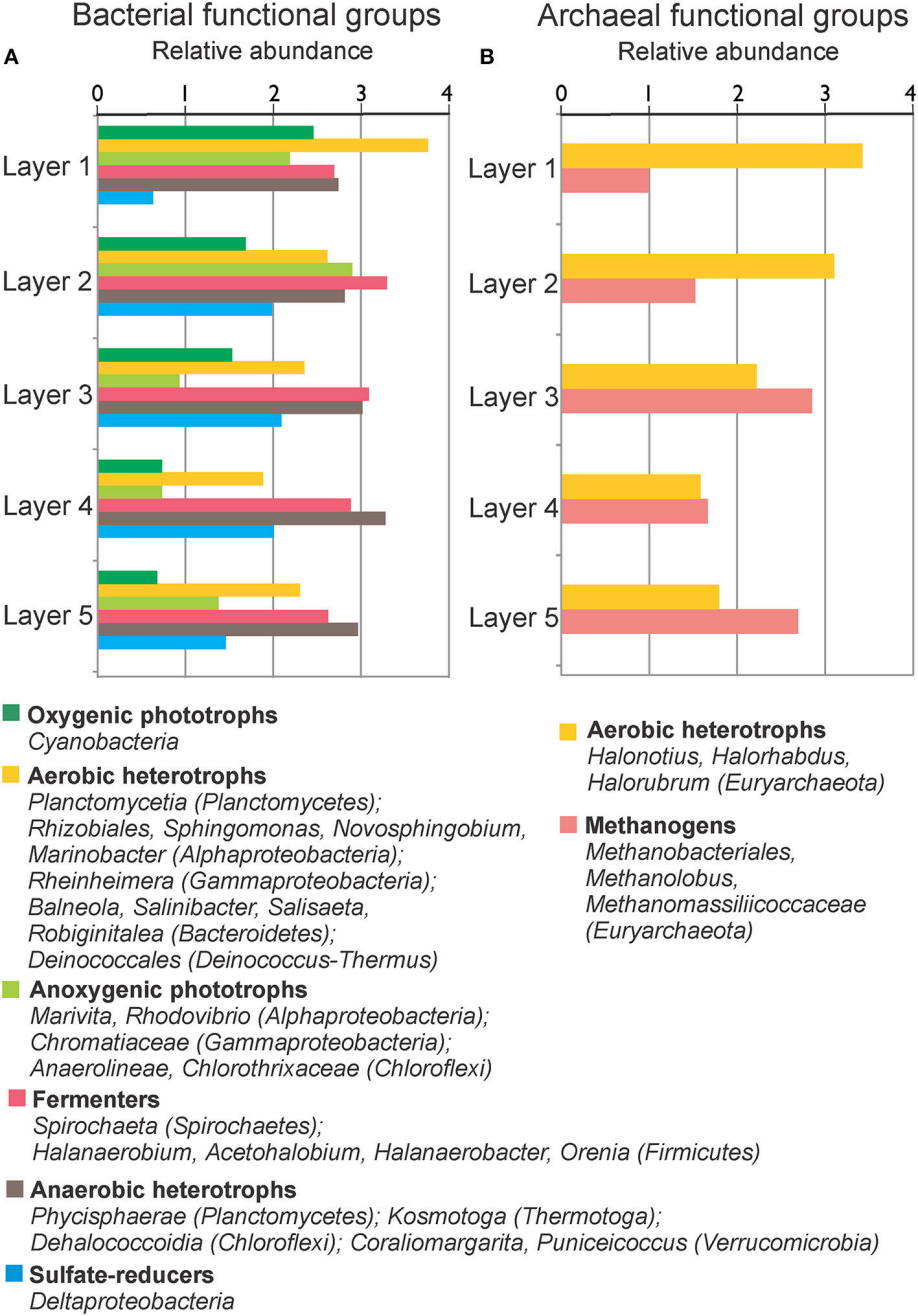
**Relative abundance (log 10 base) of bacterial (A) and archaeal (B) functional groups by layers in MA1**. Functional groups are formed with the available metabolic information from the microorganisms present in the sample.

The OTUs in the different layers of MA1 could be classified to the phylum level (Figure 6). Commonly shared phyla in the five mat layers included *Deinococcus-Thermus, Euryarchaeota, Bacteroidetes, Planctomycetes, Firmicutes, Proteobacteria, Spirochaetes, Chloroflexi*, and candidate division BRC1. *Euryarchaeota* was the most abundant phylum followed by *Planctomycetes*, especially in the three deepest layers. In the top two layers the euryarchaeotal OTUs were mainly assigned to the class *Halobacteria*. In contrast, in the two bottom layers the euryarchaeotal OTUs were principally designated to the classes *Methanobacteria* and *Thermoplasmata*, distantly followed by *Methanomicrobia* (*Methanolobus*) (Table S3; Figure 7B). In the uppermost two layers, some OTUs related to *Halobacteria* with more than 1% 16S rRNA sequences could be classified at genus level as *Halonotius, Halorhabdus*, and *Halorubrum*. The *Planctomycetes* OTUs were mainly associated to classes as *Phycisphaerae* and *Planctomycetia*. *Crenarchaeota* and to a lesser extent, *Acetothermia* were two most abundant phyla (Figure 4). OTUs for these phyla were detected in all except in the surface layer (Figure 6). *Firmicutes* were very abundant in the second and third layers, with OTUs classified within the family *Halanaerobiaceae* (Class *Clostridia*) and the genera *Halanaerobium* and *Halanaerobacter* (Table S3; Figure 7A). *Chloroflexi* were found in the highest amounts in the second and fourth layers with OTUs belonging to the classes *Anaerolineae* and *Chloroflexia* (Table S3). *Deinococcus-Thermus* (with OTUs related to family *Trueperaceae*) were abundant in the surface layer of MA1. The presence of *Cyanobacteria* (OTUs related to family *Cyanobacteraceae*) decreased sharply from the surface to the second layer, and was undetectable in the deeper layers (Figures 6, 7A). A high proportion of OTUs in the top layer could be classified within *Bacteroidetes*, especially to the class *Rhodothermi* and the genus *Salisaeta*. Similarly, *Verrucomicrobia* OTUs assigned to the class *Opitutae* were present in relatively high numbers in the surface layer as well (Table S3; Figure 7A). Compared to other layers, a larger number of OTUs attributed to *Gracilibacteria, Spirochaetes* and candidate division BCR1 were present in the second and fourth layers. The number of OTUs that could not be assigned to any taxon increased with depth.

**Figure 8:**
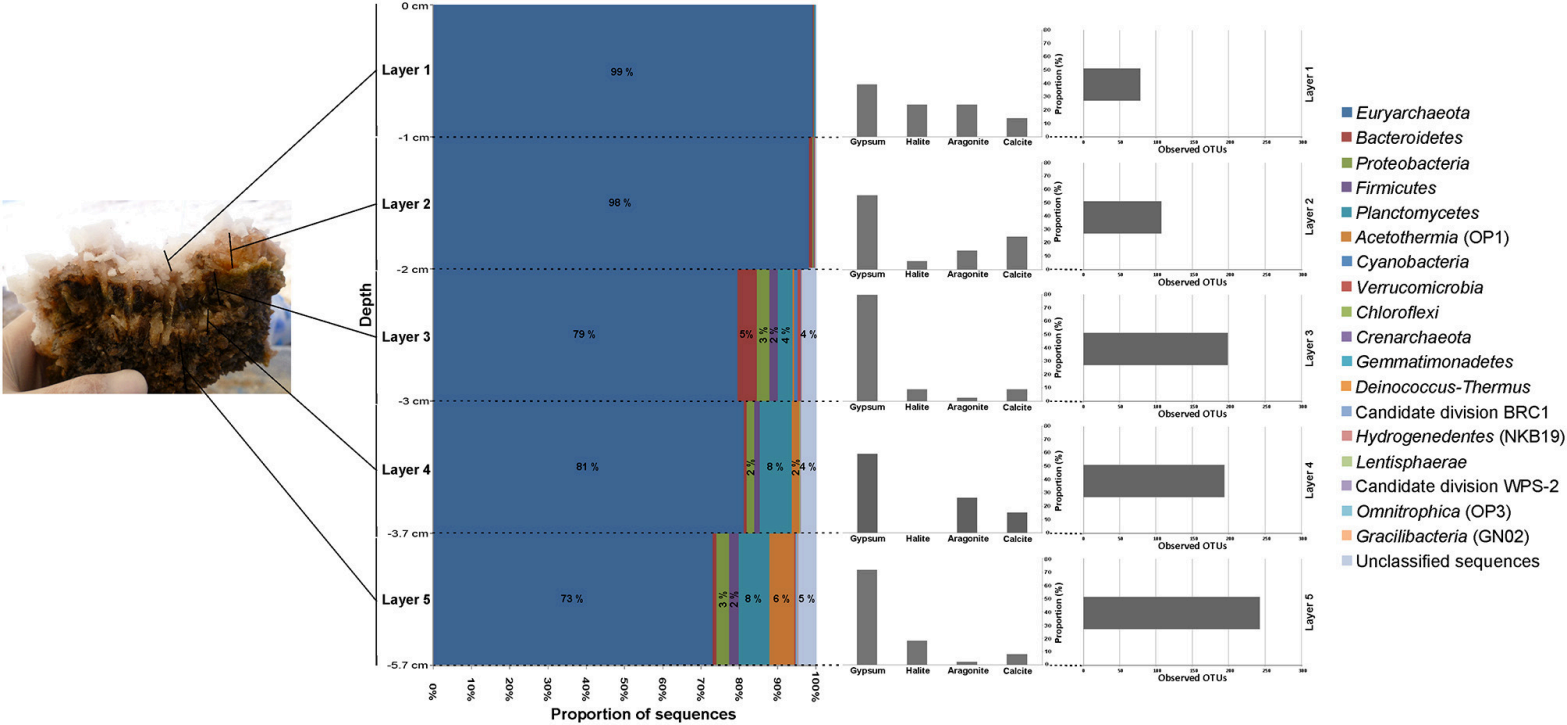
**Taxonomic composition by layers in EVD**. Sequences were assigned taxonomically using Greengenes database with a minimum percentage similarity of 97% and a minimum *e*-value of 10^−5^.

**Figure 9:**
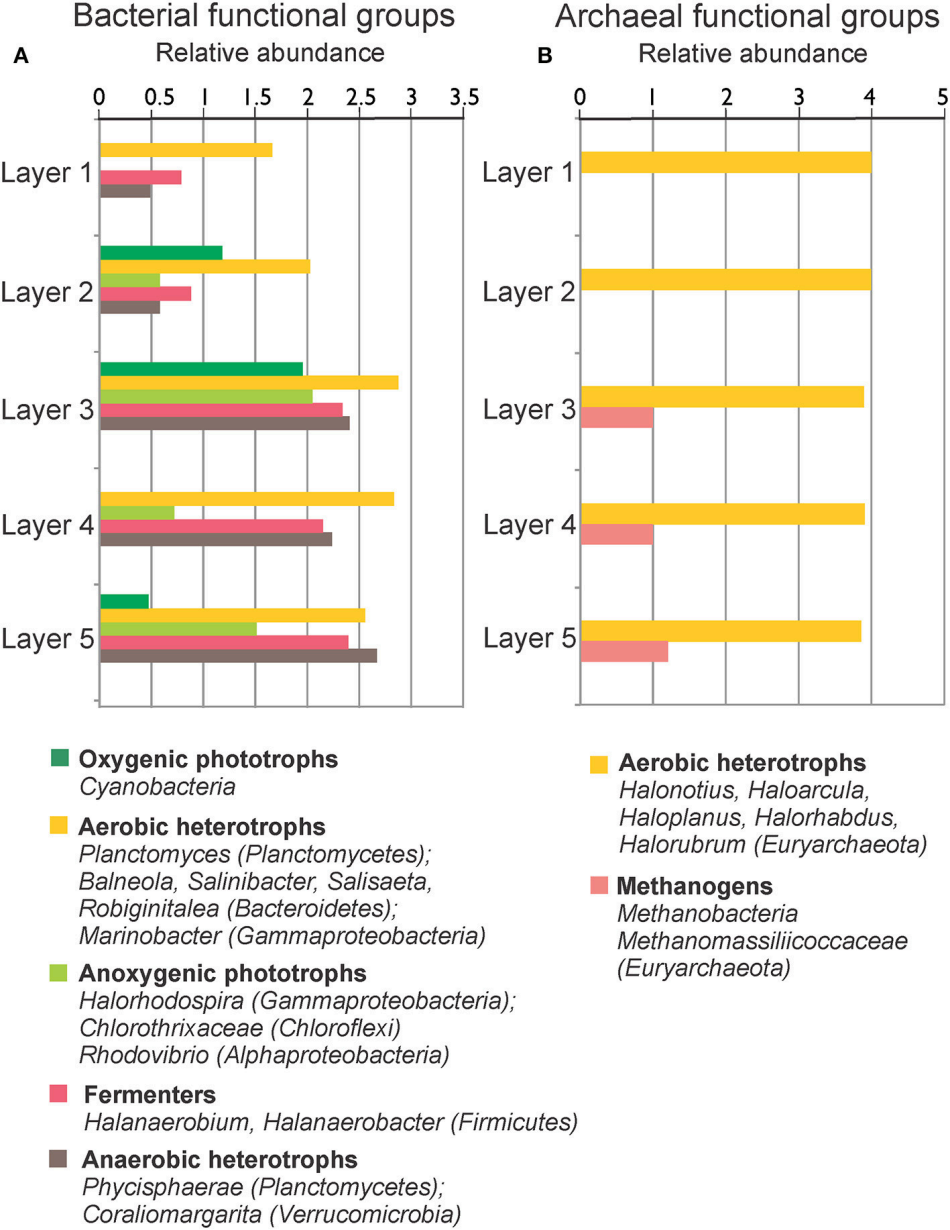
**Relative abundance (log 10 base) of bacterial (A) and archaeal (B) functional groups by layers in EVD**. Functional groups are formed with the available metabolic information from the microorganisms present in the sample.

EVD displayed a limited diversity and dominance at phylum level (Figure 8). The phylum *Euryarchaeota* was ubiquitous in all layers, particularly in the first and second layers. This phylum was almost exclusively composed of the class *Halobacteria* (Figure 9B). In all layers of EVD, a large proportion of the total 16S rRNA sequences was assigned to the genus *Halonotius* (58–24%), followed by minor genera such as *Halorhabdus, Halorubrum*, or *Haloarcula* (Table S4). A minor proportion of OTUs classified within other phyla: low proportions of 16S rRNA sequences belonging to *Bacteroidetes, Proteobacteria*, and *Firmicutes* were detected in all layers. The highest representation of *Bacteroidetes* was found in the third layer, although in the second and the third layers some OTUs classified within the genus *Salinibacter* (Table S4), and *Proteobacteria* (OTUs classified within the class *Gammaproteobacteria*) and *Firmicutes* (OTUs belonging to the class *Clostridia* and orders *Thermoanaerobacterales* and *Halanaerobiales*) were more abundant in the bottom layers [from middle (third) layer to the bottom (fifth) layer; Table S4; Figure 9A]. *Planctomycetes* was also observed in the three deepest layers, with OTUs associated to the orders *Phycisphaerales, Pirellulales*, and *Planctomycetales* (the genus *Planctomyces* is assigned to this latter order), and *Acetothermia* in the two deepest layers. Similar to MA1 as noted above, the proportion of unclassified OTUs increased with depth in EVD.

## Discussion

Contemporary microbial mats, including microbialites, typically thrive in extreme environments such as High Altitude Andean Lakes (HAAL). Several of the HAAL ecosystems have been described (Thiel et al., 2010; Farías et al., 2013; Gomez et al., 2014; Rasuk et al., 2016), but relatively little is known about the microbial diversity of the community in relation to biogeochemistry, especially that of the benthic ecosystems. The current investigation is the first in which the total microbial diversity was evaluated and interpreted in relation to the geochemical and physicochemical characteristics. The comparison of different types of benthic microbial ecosystems (e.g., with varying degrees of lithification) in Laguna Tebenquiche, and also an in-depth analysis of separate depth horizons in two contrasting laminated systems unambiguously revealed that archaea comprise the bulk of the microbial diversity. Previous diversity studies in benthic ecosystems of HAAL using high-throughput genome sequencing focused on the bacterial composition only (Farías et al., 2014). Furthermore, the archaeal and bacterial diversity in the water column of several HAAL including Laguna Tebenquiche was determined using DGGE in combination with specific primers for each Domain (Demergasso et al., 2004). A high-throughput genomic sequencing approach also deploying different archaeal and bacterial primers was used in a lithifying microbial mat from a hypersaline lake in Kiribati (i.e., not a HAAL) and revealed that the bacterial diversity was approximately three times higher than archaeal (Schneider et al., 2013). Thus, to our knowledge, an accurate assessment of the relative contribution of archaea to the total community composition in any hypersaline microbial sedimentary system was lacking until the current study.

A variety of benthic microbial ecosystems (e.g., mats, rhizome-associated concretions, and endoevaporites) showing an increased degree of lithification developed along a salinity gradient in Laguna Tebenquiche. Archaea were dominant in all five sedimentary structures, with *Euryarchaeota* the single largest contributing phylum and the presence of *Crenarchaeota* reported for first time in HAAL ecosystems. Euryarchaeal OTUs comprised 62 and 97% of the total diversity in MA2 and EVD, respectively, which represent the sites at with the highest conductivity. The class *Halobacteria*, which organisms require Na^+^ for growth and therefore can be expected to thrive in hypersaline Laguna Tebenquiche (Grant, 2001; Oren, 2010), was a major contributor to this phylum. The water column of Laguna Tebenquiche contains a high arsenic concentration (0.07 mM; Table S1), which can promote halobacterial growth. A potential role for As cycling was documented in biofilms of another hypersaline, alkaline HAAL, Laguna Diamante, where halobacterial abundance reached 94% of total OTUs (Rascovan et al., 2016). Analysis of the Diamante biofilm metagenome revealed that genes for As(III) oxidation and As(V) reduction were ubiquitous, indicating that As cycling supported energy conservation (Rascovan et al., 2016). Similar to Laguna Diamante, we postulate that *Halobacteria* inhabiting Laguna Tebenquiche could use arsenic for bioenergetics purposes.

MA1, RAC1, and RAC2 contained euryarchaeal OTUs with gene sequences similar to those of methanogenic archaea from the class *Methanobacteria*, the family *Methanomassiliicoccaceae* (class *Thermoplasmata*) and the candidate division MSBL1. 16S rRNA sequences and fosmids of the uncultured candidate division MSBL1 have been retrieved from hypersaline anoxic environments such as the deep-sea hypersaline anoxic brines in the Mediterranean Sea (van der Wielen et al., 2005; Daffonchio et al., 2006; Borin et al., 2009; Yakimov et al., 2013) and a hypersaline microbial mat of a solar saltern (López-López et al., 2013). The majority of archaeal 16S rRNA sequences recovered from these ecosystems belonged to the uncultured candidate division MSBL1 and combined methane production that was observed suggests that this archaeal lineage is involved in methanogenesis at extreme salinities. Methane production has been observed in MA1 samples (Visscher, unpublished), validating a potential biogeochemical role for MSBL1-like organisms in Laguna Tebenquiche.

The most abundant euryarchaeal OTUs in RAC1, MA1, and MA2 were classified into the deep-sea hydrothermal vent Euryarchaeotic group 1 (DHVEG-1). The uncultured DHVEG-1 members were found in the water column and sediment of anoxic deep-sea hydrothermal vents no additional information is available (Takai and Horikoshi, 1999).

The crenarchaeal OTUs make up 4–17% of the sequences in mats and rhizome-associated concretions. Many of these OTUs could be classified as MBGB, the biogeochemical function of which is highly speculative, but, based on observations in hypersaline microbial mats, could involve sulfate reduction (Robertson et al., 2009).

In contrast to earlier work (Demergasso et al., 2008; Farías et al., 2014) that reported predominance of ubiquitous *Bacteroidetes* and *Proteobacteria* in Laguna Tebenquiche, the current study found that these phyla were not dominant. Instead, *Plantomycetes, Firmicutes*, and *Acetotermia* were the main contributors to bacterial diversity. This finding could result from primer bias: earlier studies (5,7) focused on the bacterial community, whereas the current investigation analyzed the total microbial community (bacteria and archaea). OTUs belonging to *Planctomycetes*, which was the most abundant phylum within the bacteria in MA1 and MA2 and the second and third-most abundant phylum in RAC1 and RAC2, respectively, were mainly associated with the class *Phycisphaerae*. This class was recently described by one cultured and several uncultured representatives retrieved from marine environments and soils (Fukunaga et al., 2009). Diverse organoheterotrophic capabilities allows organisms belonging to this class to colonize of a wide variety of ecosystems, ranging from aquatic to terrestrial habitats including several extreme environments. *Phycisphaerae* were previously found in microbial mats of hypersaline lakes in the Bahamas (Baumgartner et al., 2009a) and Kiribati (Schneider et al., 2013).

The OTUs assigned to the phylum *Firmicutes*, which were prevalent in RAC2 (Figure 4), comprised mainly of fermenter halophilic anaerobic members of the order *Halanaerobiales*. OTUs assigned to the phylum *Acetothermia*, especially the taxon KB1, were ubiquitous in RAC2 (25% of total sequences) and less in both mats (4–5% of total sequences; Figure 4). Members of this phylum are uncultivated thermophiles (Hugenholtz et al., 1998; Costa et al., 2009; Kim et al., 2012; Németh et al., 2014) and the partial reconstructed genome indicates that the reductive acetyl-CoA pathway is used for CO_2_ fixation. It has been suggested that members of this phylum contribute significantly to primary production under anoxic oligocarbophylic conditions (Takami et al., 2012). It should be noted that mats are organic rich, but the bulk of this organic matter is made up by the complex structure of expolymeric substances (EPS) (Decho, 2000; Decho et al., 2005). The majority of this EPS is recalcitrant, notably in deeper layers, resisting microbial degradation (Braissant et al., 2009). As a result, especially the anoxic parts of EPS-rich mats and microbialites could be deprived of readily available organic substrates for respiration, thereby increasing the importance of anaerobic CO_2_ fixation and methanogenesis in these systems.

Typically, cyanobacteria are the principle autotrophs in microbial mats (Visscher and van Gemerden, 1993; Visscher and Stolz, 2005). However, given the limited presence of cyanobacterial as well as proteobacterial OTUs in our samples, it is plausible that, in addition to the *Halobacteria* discussed above, other organisms such as *Acetothermia* contribute to community CO_2_ fixation in Tebenquiche. The scarcity of cyanobacteria in the current study corroborates earlier observations of a low cyanobacterial presence in mats and evaporites from the Salar de Atacama (Farías et al., 2013, 2014; Rasuk et al., 2014, 2016). However, we cannot rule out that the cyanobacterial community comprises a few species with high abundance, and high metabolic activity. Similar observations of low cyanobacterial diversity were made previously by other investigators in a variety of mats and microbialites (McKay et al., 2003; Ley et al., 2006; Baumgartner et al., 2009a,b; Lynch et al., 2012). *Alphaproteobacteria* and *Gammaproteobacteria*, prevalent in MA1 and EVD, are typical inhabitants of microbial mats and microbialites (Dupraz et al., 2011) and may significantly contribute to primary production (van Gemerden, 1993). The depth profiles of sulfide (Figure 3) support the notion that sulfide-oxidizing bacteria, including both chemolithoautotrophs and photolithoautrophs, could also contribute to the organic carbon pool.

OTUs belonging to *Chlorothixaceae* and *Anaerolinaeae* (*Chloroflexi*), *Rhodovibrio* (*Alphaproteobacteria*), and purple sulfur bacteria classified into the family *Chromatiaceae* and *Ectothiorhodospiraceae* (*Gammaproteobacteria*) were found and most likely carrying out the anoxygenic photosynthesis in Laguna Tebenquiche.

Members of *Bacteroidetes* were present but scarce in all samples. *Bacteroidetes* are believed to be among the best-adapted organisms to growth under the wide range of physicochemical conditions found in Atacama Desert (Demergasso et al., 2004). In addition, this phylum was observed in a variety of other hypersaline systems including microbial mats (Sørensen et al., 2005; Ley et al., 2006), water and sediment samples (Demergasso et al., 2004, 2008, 2010; Dorador, 2007), and evaporites (Stivaletta et al., 2011; Farías et al., 2014; Rasuk et al., 2014).

The presence of the phylum *Verrucomicrobia* was strongly correlated to RAC1. In similar microbialites found under less saline conditions [e.g., stromatolites from Laguna Socompa (Argentina) and a microbialites in Laguna La Brava in Atacama Desert (Chile)], with a conductivity of less than conductivity 115 mS/cm (Farías et al., 2013, 2014), OTUs related to this phylum were also found. It is plausible that members of this phylum are ubiquitous in microbialites found in high altitude whenever the salinity is not excessively high.

To further investigate the impact of the limited cyanobacterial diversity discussed above and unusually high abundance of archaea, we determined the microbial diversity in discrete depth horizons.

*Deinococcus* sp. has been a model organism for investigating UV resistance (Arrage et al., 1993), and is frequently found in a variety of microbial mats (Skirnisdottir et al., 2000; Pagaling et al., 2012; Abed et al., 2014; Tytgat et al., 2014), including stromatolites of Shark Bay, Australia (Goh et al., 2009). In the non-lithifying mat MA1, a large proportion of 16S rRNA sequences in the surface layer were associated with the phylum *Deinococcus*-*Thermus*. Farías et al. (2013) suggested that elevated levels of UV radiation associated with high altitude ecosystems limit microbial abundance and diversity, e.g., in stromatolites found in Laguna Socompa (Argentina). Therefore, the high dominance of the phylum *Deinococcus*-*Thermus* in the surface layer was likely due to their UV resistance mechanism. We also found that crenarchaeal and acetothermial OTUs that classified as MBGB and KB1, respectively, both prevalent in deeper layers, were absent in the surface layer of MA1 where the oxygen concentrations peaked (Table S3).

Some metabolic quandaries could be addressed through analysis in individual layers of MA1 and EVD. As outlined above, the cyanobacterial contribution to primary production appeared limited. Anoxygenic phototrophic *Alphaproteobacteria* and *Gammaproteobacteria* and *Chloroflexi* were present in the surface and second layers of MA1 and in the second and third layers of EVD, immediately underneath the surface halite layer. This distribution was further substantiated by microelectrode measurements of oxygen distribution (Figure 3), showing a maximum concentration at 1.5–1.75 mm of depth in MA1 (corresponding to surface and second layer) and between 6 and 8 mm of depth in EVD (coinciding with the second and third layers). Some gammaproteobacterial OTUs in both MA1 and EVD were related to the order *Chromatiales* (*Gammaproteobacteria*), which has representatives containing bacteriochlorophyll a and b. Some of these *Chromatiales* grow under both oxic and anoxic conditions and are capable of photolithoautotrophic and chemolithoautotrophic metabolism (van Gemerden, 1993).

Both aerobic and anaerobic heterotrophic microorganisms were detected in MA1 and EVD. The main heterotrophs in all layers of EVD were aerobic haloarchaea. Euryarchaeal OTUs related to anaerobic and methanogenic archaea were found in MA1 and EVD, with increasing proportions of 16S rRNA gene sequences with increasing depth, where permanently anoxic conditions prevail. A plethora of anaerobic fermenters resided in all layers of MA1 and EVD, however mainly represented by *Firmicutes*. Sulfate reduction was likely carried out by *Deltaproteobacteria* in MA1, including in the surface and second layers, both of which are oxic. Studies performed in several microbial mats, including hypersaline mats in Guerrero Negro, Solar Lake, Kiritimati Atoll, Shark Bay, Texel, and the Bahamas revealed high sulfate reduction rates in oxic layers as well (Canfield and Des Marais, 1991; Fründ and Cohen, 1992; Visscher et al., 1992, 1998; Bühring et al., 2009; Pages et al., 2014; Wong et al., 2015).

Most diversity studies in microbial mats and microbialites to date focused only on bacteria and showed important contributions of *Cyanobateria, Proteobacteria*, and *Bacteroidetes* to the total diversity in these system. Although primer bias, mentioned above, clearly impact the outcome of molecular investigations, the primacy of bacteria over archaea has been reported in numerous benthic microbial ecosystems, including hypersaline mats of Kribati (Schneider et al., 2013), open marine stromatolites (Baumgartner et al., 2009a), and hypersaline microbialites in the Bahamas (Baumgartner et al., 2009b) and Cuatro Cienegas, Guerrero Negro, and Shark Bay (Burns et al., 2004; Ley et al., 2006; Valeria et al., 2007; Demergasso et al., 2008). The high relative abundance of archaea in the samples from Laguna Tebenquiche warrants a reassessment of the role of archaea in these benthic microbial ecosystems and necessitates a similar approach when investigating other microbial mats.

## Conclusion

In conclusion, in Laguna Tebenquiche, *Euryarchaeota* was one of the most abundant phyla in all samples studied, notably in EVD, in which it represented 97% of the 16S rRNA sequences. Most of these euryarchaeal OTUs were classified within the class *Halobacteria* or anaerobic and methanogenic archaea. *Halobacteria* mainly grow by aerobic oxidation of amino acids, carbohydrates or alcohol (i.e., glycerol), but can also photophosphorylate under anoxic conditions (Hartmann et al., 1980). This suggests an important role for (an)aerobic heterotrophy and potentially methanogenesis carried out by *Euryarchaeota* in these benthic ecosystems. *Planctomycetes* played also a key role in mats and rhizome-associated concretion samples, notably the class *Phycisphaerae*, which are aerobic organoheterotrophs. In addition to cyanobacterial primary production, anoxygenic photosynthesis by *Chromatiales*, possibly *Chloroflexi* and the candidate family *Chlorotrichaceae* (in RAC1 and mat samples) could contribute to CO_2_ fixation in the mat. Other taxa present that could play a metabolic role include the uncultured candidate division MSBL1, possibly be involved in methanogenesis, and the uncultured MBGB. Some members of *Proteobacteria* could contribute to sulfate reduction and the uncultured phylum *Acetothermia* could perform CO_2_ fixation through the reductive acetyl-CoA pathway. The importance of understanding the taxonomic and metabolic diversity in Laguna Tebenquiche resides in the extreme conditions of this environment, which may provide new insights into microbial processes in the Earth's early history and potentially on habitable exoplanets.

## Author contributions

AF performed data analysis, interpreted data and wrote paper. MR contributed in sampling and data analysis. PV contributed in work proposal, sampling, performed oxygen and sulfide profile analysis and wrote paper. MC, FN obtained funding for the original project idea and performed physicochemical analysis. DP performed mineral analysis. MP contributed in oxygen and sulfide profile analysis. AV contributed in data analysis. MF obtained funding for the original project idea, contributed in work proposal, sampling and wrote paper. All authors read and approved this manuscript.

### Conflict of interest statement

The authors declare that the research was conducted in the absence of any commercial or financial relationships that could be construed as a potential conflict of interest.

## Supplementary material

Figure S1 **Rarefaction curves plotting the number of observed OTUs as a function of the number of sequences in microbial mats (MA1 and MA2), rhizome-associated lithified concretions (RAC1 and RAC2) and evaporite (EVD)**.

Figure S2 **Rarefaction curves plotting the number of observed OTUs as a function of the number of sequences. (A)** Rarefaction curves by layers in MA1. **(B)** Rarefaction curves by layers in EVD.

Table S1 **Physico-chemical parameters for the overlying water from the different samples studied**.

Table S2 **Observed microbial richness and diversity estimates based on 97% OTU clusters in microbial mats (MA1 and MA2), rhizome-associated lithified mats (RAC1 and RAC2) and evaporite (EVD)**.

Table S3 **Abundant microbial OTUs classified at the lowest possible taxonomic level in MA1**. Each OTU contains at less 1% 16S rRNA sequences.

Table S4 **Abundant microbial OTUs classified at the lowest possible taxonomic level in EVD**. Each OTU contains at less 1% 16S rRNA sequences.
