# Supplementary figures and images for: Microbial Diversity in Sediment Ecosystems (Evaporites Domes, Microbial Mats, and Crusts) of Hypersaline Laguna Tebenquiche, Salar de Atacama, Chile
Source: Front Microbiol. 2016 Aug 22;7:1284. doi: 10.3389/fmicb.2016.01284 (PMC4992683; doi:10.3389/fmicb.2016.01284)

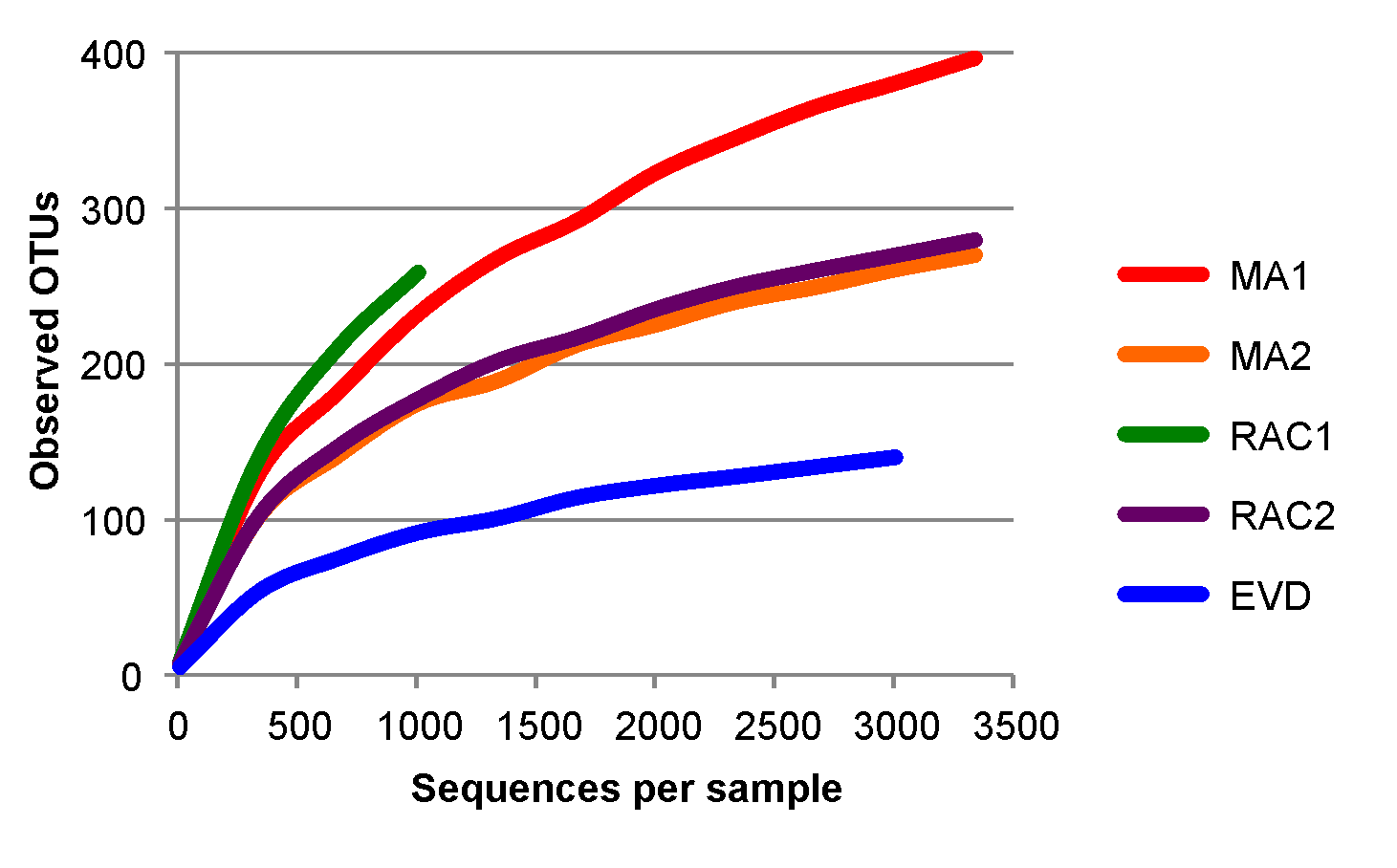

Supplement: Figure S1 — Rarefaction curves plotting the number of observed OTUs as a function of the number of sequences in microbial mats (MA1 and MA2), rhizome-associated lithified concretions (RAC1 and RAC2) and evaporite (EVD). [file Image1.TIF]

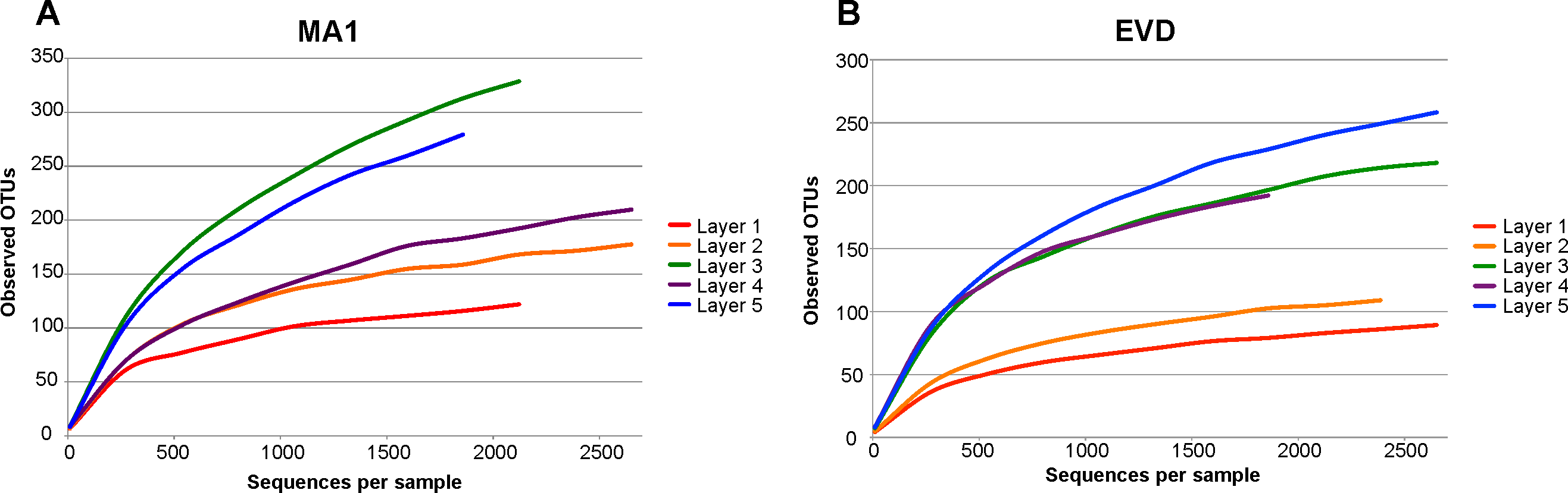

Supplement: Figure S2 — Rarefaction curves plotting the number of observed OTUs as a function of the number of sequences. (A) Rarefaction curves by layers in MA1. (B) Rarefaction curves by layers in EVD. [file Image2.TIF]
