# Supplementary material for: Microbial Diversity in Sediment Ecosystems (Evaporites Domes, Microbial Mats, and Crusts) of Hypersaline Laguna Tebenquiche, Salar de Atacama, Chile
Source: Front Microbiol. 2016 Aug 22;7:1284. doi: 10.3389/fmicb.2016.01284 (PMC4992683; doi:10.3389/fmicb.2016.01284)
Supplement: Table S1 — Physico-chemical parameters for the overlying water from the different samples studied. [file Table1.DOCX]

**Table S1.** Physico-chemical parameters for the overlying water from the different samples studied.

| **Physico-chemical parameter** | **Unit** | **MA1** | **MA2** | **RAC1** | **EVD** |
| --- | --- | --- | --- | --- | --- |
| Macroelements |  |  |  |  |  |
| Total organic Carbon | mg/L | 9 | 9 | 14 | 10 |
| Dissolved oxygen | mg/L | 5.8 | 3.7 | 7.8 | 6.1 |
| Nitrate | µg/L | 1,100 | 1,954 | 777 | 1,314 |
| Nitrite | µg/L | <0.2 | 1.2 | <0.2 | 1.2 |
| Total organic Nitrogen | µg/L | 823 | 755 | 885 | 720 |
| Total Phosphorus | ug/L | 1,045 | 1,438 | 678 | 1,190 |
| Orthophosphate | µg/L | 104 | 18 | 627 | 81 |
| Sulphate | mg/L | 14,671 | 33,787 | 8,480 | 16,167 |
| Sulphur | mg/L | 4,897 | 11,278 | 2,831 | 5,396 |
| Total Sulphide | mg/L | < 0.2 | < 0.2 | < 0.2 | < 0.2 |
| Microelements |  |  |  |  |  |
| Sodium | mg/L | 47,110 | 95,930 | 17,585 | 54,230 |
| Chloride | mg/L | 75,090 | 175,648 | 31,135 | 84,934 |
| Potassium | mg/L | 4,356 | 9,923 | 2,385 | 5,031 |
| Magnesium | mg/L | 3,109 | 7,471 | 1,205 | 3,520 |
| Calcium | mg/L | 845 | 412 | 1,073 | 870 |
| Dissolved Boron | mg/L | 144 | 402 | 115 | 161 |
| Total Boron | mg/L | 318 | 543 | 158 | 363 |
| Dissolved Lithium | mg/L | 201 | <0.004 | 98 | 231 |
| Total Lithium | mg/L | 352 | 404 | 145 | 445 |
| Silica | mg/L | 47 | 19 | 51 | 46 |
| Dissolved Arsenic | mg/L | 3 | 2 | 2 | 3 |
| Total Arsenic | mg/L | 4 | 5 | 3 | 4 |
| Aluminium dissolved | mg/L | <0.15 | <0.15 | <0.15 | <0.15 |
| Total Aluminium | mg/L | 0 | 0 | 0 | 0 |
| Dissolved Barium | mg/L | 0.046 | <0.001 | 0.068 | 0.046 |
| Total Barium | mg/L | 0.049 | 0.007 | 0.107 | 0.050 |
| Dissolved Beryllium | mg/L | <0.01 | <0.01 | <0.01 | <0.01 |
| Total Beryllium | mg/L | <0.01 | <0.01 | <0.01 | <0.01 |
| Dissolved Cadmium | mg/L | <0.001 | <0.001 | <0.001 | <0.001 |
| Total Cadmium | mg/L | <0.001 | <0.001 | <0.001 | <0.001 |
| Dissolved Cobalt | mg/L | <0.001 | <0.001 | 0.001 | <0.001 |
| Total Cobalt | mg/L | 0.021 | 0.016 | 0.010 | 0.017 |
| Copper Dissolved | mg/L | <0.003 | 0.003 | 0.004 | 0.008 |
| Copper Total | mg/L | 0.009 | 0.008 | 0.008 | 0.009 |
| Dissolved Chromium | mg/L | <0.002 | <0.002 | <0.002 | <0.002 |
| Total Chromium | mg/L | 0.006 | 0.004 | 0.006 | 0.002 |
| Dissolved Iron | mg/L | 0.004 | 0.003 | 0.010 | 0.005 |
| Total Iron | mg/L | 0.060 | 0.027 | 0.052 | 0.016 |
| Dissolved Manganese | mg/L | 0.401 | 0.133 | 0.337 | 0.383 |
| Total Manganese | mg/L | 0.468 | 0.242 | 0.846 | 0.518 |
| Dissolved Molybdenum | mg/L | 0.040 | 0.019 | <0.003 | 0.041 |
| Total Molybdenum | mg/L | 0.059 | 0.061 | 0.038 | 0.047 |
| Dissolved Nickel | mg/L | 0.007 | <0.003 | <0.003 | 0.006 |
| Total Nickel | mg/L | 0.036 | 0.005 | 0.011 | 0.051 |
| Dissolved Silver | mg/L | 0.04 | 0.06 | <0.01 | 0.04 |
| Total Silver | mg/L | 0.13 | 0.17 | 0.02 | 0.13 |
| Dissolved Lead | mg/L | <0.008 | <0.008 | <0.008 | <0.008 |
| Total Lead | mg/L | <0.008 | <0.008 | <0.008 | <0.008 |
| Dissolved Selenium | mg/L | <0.02 | <0.02 | <0.02 | <0.02 |
| Total Selenium | mg/L | <0.02 | 0.09 | <0.02 | <0.02 |
| Dissolved Zinc | mg/L | <0.002 | 0.054 | 0.047 | 0.032 |
| Total Zinc | mg/L | 0.071 | 0.097 | 0.125 | 0.063 |
