# Supplementary material for: Microbial Diversity in Sediment Ecosystems (Evaporites Domes, Microbial Mats, and Crusts) of Hypersaline Laguna Tebenquiche, Salar de Atacama, Chile
Source: Front Microbiol. 2016 Aug 22;7:1284. doi: 10.3389/fmicb.2016.01284 (PMC4992683; doi:10.3389/fmicb.2016.01284)
Supplement: Table S2 — Observed microbial richness and diversity estimates based on 97% OTU clusters in microbial mats (MA1 and MA2), rhizome-associated lithified mats (RAC1 and RAC2) and evaporite (EVD). [file Table2.DOCX]

**Table S2.** Observed microbial richness and diversity estimates based on 97% OTU clusters in microbial mats (MA1 and MA2), rhizome-associated lithified mats (RAC1 and RAC2) and evaporite (EVD).

| **Sample** | **Number of total reads** | **Seqs/Sample** | **Observed OTUs** | **Chao1** | **Shannon** | **Equitability** | **Dominance** | **Simpson** |
| --- | --- | --- | --- | --- | --- | --- | --- | --- |
| MA1 | 4534 | 1070 | 240 | 531 | 6.24 | 0.790 | 0.040 | 0.960 |
| MA2 | 3345 | 1070 | 169 | 240 | 5.69 | 0.769 | 0.053 | 0.947 |
| RAC1 | 1070 | 1070 | 266 | 407 | 6.55 | 0.814 | 0.037 | 0.963 |
| RAC2 | 3357 | 1070 | 188 | 342 | 5.66 | 0.750 | 0.059 | 0.941 |
| EVD | 3132 | 1070 | 96 | 128 | 3.00 | 0.456 | 0.405 | 0.595 |
