# Supplementary material for: Microbial Diversity in Sediment Ecosystems (Evaporites Domes, Microbial Mats, and Crusts) of Hypersaline Laguna Tebenquiche, Salar de Atacama, Chile
Source: Front Microbiol. 2016 Aug 22;7:1284. doi: 10.3389/fmicb.2016.01284 (PMC4992683; doi:10.3389/fmicb.2016.01284)
Supplement: Table S3 — Abundant microbial OTUs classified at the lowest possible taxonomic level in MA1. Each OTU contains at less 1% 16S rRNA sequences. [file Table3.DOCX]

**Table S3**. Abundant microbial OTUs classified at the lowest possible taxonomic level in MA1. Each OTU contains at less 1% 16S rRNA sequences.

| Layer 1 (0-1.5 mm) | | Layer 2 (1.5-4 mm) | | Layer 3 (4-6.5 mm) | | Layer 4 (6.5-9.5 mm) | | Layer 5 (9.5-11.5 mm) | |
| --- | --- | --- | --- | --- | --- | --- | --- | --- | --- |
| GBI-58  (Phyl. *Deinococcus-Thermus*/  Fam. *Trueperaceae*) | 37.3 | **Gen. *Halanaerobacter***  (Phyl. *Firmicutes*/  Class *Clostridia*) | 17.4 | **DHVEG-1**  (Phyl. *Euryarchaeota*/  Class *Thermoplasmata*) | 26.3 | **MBGB**  (Phyl. *Crenarchaeota*) | 21.4 | **MBGB**  (Phyl. *Crenarchaeota*) | 22.8 |
| Fam. *Halobacteriaceae*  (Phyl. *Euryarchaeota*/  Class *Halobacteria*) | 8.6 | **MBGB**  (Phyl. *Crenarchaeota*) | 15.9 | **MBGB**  (Phyl. *Crenarchaeota*) | 7.2 | **DHVEG-1**  (Phyl. *Euryarchaeota*/  Class *Thermoplasmata*) | 21.0 | **DHVEG-1**  (Phyl. *Euryarchaeota*/  Class *Thermoplasmata*) | 22.0 |
| Gen. *Halorubrum*  (Phyl. *Euryarchaeota*/  Class *Halobacteria*) | 8.6 | **3BR-5F**  (Phyl. *Gracilibacteria*) | 8.5 | **Fam. *Halanaerobiaceae***  (Phyl. *Firmicutes*/  Class *Clostridia*) | 7.2 | **AKAU3564**  (Phyl. *Planctomycetes*/  Class *Phycisphaerae*) | 15.5 | **AKAU3564**  (Phyl. *Planctomycetes*/  Class *Phycisphaerae*) | 4.9 |
| Gen. *Salisaeta*  (Phyl. *Bacteroidetes*/  Class. *Rhodothermi*) | 6.4 | **Cand. *Chlorothrix***  (Phyl. *Chloroflexi*/  Cand. fam*Chlorothrixaceae*) | 6.1 | **AKAU3564**  (Phyl. *Planctomycetes*/  Class *Phycisphaerae*) | 6.7 | **OPB11**  (Phyl. *Chloroflexi*/  Class *Anaerolineae*) | 6.8 | **KB1**  (Phyl. *Acetothermia*) | 4.3 |
| Fam. *Rhodothermaceae*  (Phyl. *Bacteroidetes*/  Class. *Rhodothermi*) | 5.3 | **AKAU3564**  (Phyl. *Planctomycetes*/  Class *Phycisphaerae*) | 4.6 | **KB1**  (Phyl. *Acetothermia*) | 5.4 | **3BR-5F**  (Phyl. *Gracilibacteria*) | 4.5 | **Uncultured soil bacterium PRR-12**  (Phyl. *Latescibacteria*) | 2.7 |
| Gen. *Halanaerobium*  (Phyl. *Firmicutes*/  Class *Clostridia*) | 3.5 | **Gen. *Halorubrum***  (Phyl. *Euryarchaeota*/  Class *Halobacteria*) | 3.6 | **Fam. *Methanomassiliicoccaceae***  (Phyl. *Euryarchaeota*/  Class *Thermoplasmata*) | 3.9 | **NPL-UPA2**  (Cand. div. BRC1) | 3.0 | **MSBL9**  (Phyl. *Planctomycetes*/  Class *Phycisphaerae*) | 2.4 |
| Fam. *Pirellulaceae*  (Phyl. *Planctomycetes*/  Class *Planctomycetia*) | 3.4 | **Fam. *Halobacteriaceae***  (Phyl. *Euryarchaeota*/  Class *Halobacteria*) | 3.5 | **20c-4**  (Phyl. *Euryarchaeota*/  Class *Thermoplasmata*) | 3.6 | **MSBL9**  (Phyl. *Planctomycetes*/  Class *Phycisphaerae*) | 2.7 | **OPB11**  (Phyl. *Chloroflexi*/  Class *Anaerolineae*) | 1.9 |
| Fam. *Cyanobacteriaceae*  (Phyl. *Cyanobacteria*/  Class *Oscillatoriophycideae*) | 2.8 | **Uncultured soil bacterium PRR-11**  (Cand. div. BRC1) | 3.4 | **Gen. *Halanaerobacter***  (Phyl. *Firmicutes*/  Class *Clostridia*) | 1.8 | **Cand. div. KSB3** | 1.0 | **Fam. *Methanomassiliicoccaceae***  (Phyl. *Euryarchaeota*/  Class *Thermoplasmata*) | 1.9 |
| Fam. *Trueperaceae*  (Phyl. *Deinococcus-Thermus*/  Class *Deinococci*) | 2.5 | **Cand. div. WS1** | 2.8 | **Ord. *Methanobacteriales***  (Phyl. *Euryarchaeota*/  Class *Methanobacteria*) | 1.7 | **Uncultured soil bacterium PRR-12**  (Phyl. *Latescibacteria*) | 1.0 | **Cand. div. MSBL1**  (Phyl. *Euryarchaeota*) | 1.5 |
| Fam. *Phycisphaeraceae*  (Phyl. *Planctomycetes*/  Class *Phycisphaerae*) | 2.4 | **Fam. *Halobacteriaceae***  (Phyl. *Euryarchaeota*/  Class *Halobacteria*) | 1.9 | **B04R032**  (Cand. div. AC1) | 1.6 | **Phyl. *Planctomycetes*** | 1.0 | **20c-4**  (Phyl. *Euryarchaeota*/  Class *Thermoplasmata*) | 1.5 |
| Gen. *Halorhabdus*  (Phyl. *Euryarchaeota*/  Class *Halobacteria*) | 2.0 | **Sediment-4**  (Phyl. *Spirochaetes*/  Ord. *Leptospirales*) | 1.9 | **Cand. div. MSBL1**  (Phyl. *Euryarchaeota*) | 1.6 |  |  | **Ord. *Methanobacteriales***  (Phyl. *Euryarchaeota*/  Class *Methanobacteria*) | 1.3 |
| Gen. *Halonotius*  (Phyl. *Euryarchaeota*/  Class *Halobacteria*) | 1.7 | **Cand. fam. *Chlorothrixaceae***  (Phyl. *Chloroflexi*/  Class *Chloroflexia*) | 1.7 | **Gen. *Halanaerobium***  (Phyl. *Firmicutes*/  Class *Clostridia*) | 1.5 |  |  | **Phyl. *Planctomycetes*** | 1.2 |
| XKL75  (Phyl. *Euryarchaeota*/  Fam. *Halobacteriaceae*) | 1.6 | **Fam *Pirellulaceae***  (Phyl. *Planctomycetes*/  Class *Planctomycetia*) | 1.6 | **Fam *Pirellulaceae***  (Phyl. *Planctomycetes*/  Class *Planctomycetia*) | 1.2 |  |  | **Fam. *Halanaerobiaceae***  (Phyl. *Firmicutes*/  Class *Clostridia*) | 1.1 |
| Gen. *Coraliomargarita*  (Phyl. *Verrucomicrobia*/  Class. *Opitutae*) | 1.5 | **Gen. *Halorhabdus***  (Phyl. *Euryarchaeota*/  Class *Halobacteria*) | 1.5 | **WM88**  (Cand. div. Hyd24-12) | 1.1 |  |  | **B04R032**  (Cand. div. AC1) | 1.0 |
| Fam. *Halobacteriaceae*  (Phyl. *Euryarchaeota*/  Class *Halobacteria*) | 1.4 | **SBYZ_6080**  (Phyl. *Spirochaetes*) | 1.4 | **Uncultured soil bacterium PRR-12**  (Phyl. *Latescibacteria*) | 1.0 |  |  | **BA021**  (*Atribacteria*) | 1.0 |
| MSP41  (Phyl. *Euryarchaeota*/  Ord. *Halobacteriales*) | 1.1 | **KB1**  (Phyl. *Acetothermia*) | 1.3 | **Class *Anaerolineae***  (Phyl. *Chloroflexi*) | 1.0 |  |  | **Uncultured crenarchaeote MCG**  (Phyl. *Crenarchaeota*) | 1.0 |
|  |  | **Gen. *Salisaeta***  (Phyl. *Bacteroidetes*/  Class. *Rhodothermi*) | 1.3 |  |  |  |  |  |  |
|  |  | **Fam. *Halobacteriaceae***  (Phyl. *Euryarchaeota*/  Class *Halobacteria*) | 1.2 |  |  |  |  |  |  |
|  |  | **DHVEG-1**  (Phyl. *Euryarchaeota*/  Class *Thermoplasmata*) | 1.0 |  |  |  |  |  |  |
