# Supplementary material for: Microbial Diversity in Sediment Ecosystems (Evaporites Domes, Microbial Mats, and Crusts) of Hypersaline Laguna Tebenquiche, Salar de Atacama, Chile
Source: Front Microbiol. 2016 Aug 22;7:1284. doi: 10.3389/fmicb.2016.01284 (PMC4992683; doi:10.3389/fmicb.2016.01284)
Supplement: Table S4 — Abundant microbial OTUs classified at the lowest possible taxonomic level in EVD. Each OTU contains at less 1% 16S rRNA sequences. [file Table4.DOCX]

**Table S4.** Abundant microbial OTUs classified at the lowest possible taxonomic level in EVD. Each OTU contains at less 1% 16S rRNA sequences.

| Layer 1 (0-1 mm) | | Layer 2 (1-2 mm) | | Layer 3 (2-3 mm) | | Layer 4 (3-3.7 mm) | | Layer 5 (3.7-5.7 mm) | |
| --- | --- | --- | --- | --- | --- | --- | --- | --- | --- |
| Gen. *Halonotius*  (Phyl. *Euryarchaeota*/  Class *Halobacteria*) | 52.9 | **Gen. *Halonotius***  (Phyl. *Euryarchaeota*/  Class *Halobacteria*) | 58.0 | **Gen. *Halonotius***  (Phyl. *Euryarchaeota*/  Class *Halobacteria*) | 23.8 | **Gen. *Halonotius***  (Phyl. *Euryarchaeota*/  Class *Halobacteria*) | 18.4 | **Gen. *Halonotius***  (Phyl. *Euryarchaeota*/  Class *Halobacteria*) | 24.8 |
| Fam. *Halobacteriaceae*  (Phyl. *Euryarchaeota*/  Class *Halobacteria*) | 23.5 | **Fam. *Halobacteriaceae***  (Phyl. *Euryarchaeota*/  Class *Halobacteria*) | 12.7 | **Fam. *Halobacteriaceae***  (Phyl. *Euryarchaeota*/  Class *Halobacteria*) | 15.2 | **Gen. *Halorhabdus***  (Phyl. *Euryarchaeota*/  Class *Halobacteria*) | 13.2 | **Fam. *Halobacteriaceae***  (Phyl. *Euryarchaeota*/  Class *Halobacteria*) | 13.4 |
| Gen. *Halorhabdus*  (Phyl. *Euryarchaeota*/  Class *Halobacteria*) | 4.1 | **Gen. *Halorubrum***  (Phyl. *Euryarchaeota*/  Class *Halobacteria*) | 8.0 | **Gen. *Halorhabdus***  (Phyl. *Euryarchaeota*/  Class *Halobacteria*) | 9.2 | **Fam. *Halobacteriaceae***  (Phyl. *Euryarchaeota*/  Class *Halobacteria*) | 11.2 | **Gen. *Halorhabdus***  (Phyl. *Euryarchaeota*/  Class *Halobacteria*) | 10.9 |
| Gen. *Haloarcula*  (Phyl. *Euryarchaeota*/  Class *Halobacteria*) | 4.0 | **XKL75**  (Phyl. *Euryarchaeota*/  Fam. *Halobacteriaceae*) | 5.1 | **Gen. *Haloarcula***  (Phyl. *Euryarchaeota*/  Class *Halobacteria*) | 7.7 | **Gen. *Halorubrum***  (Phyl. *Euryarchaeota*/  Class *Halobacteria*) | 8.7 | **Gen. *Haloarcula***  (Phyl. *Euryarchaeota*/  Class *Halobacteria*) | 7.2 |
| Gen. *Halorubrum*  (Phyl. *Euryarchaeota*/  Class *Halobacteria*) | 3.6 | **Gen. *Halorhabdus***  (Phyl. *Euryarchaeota*/  Class *Halobacteria*) | 4.7 | **Gen. *Halorubrum***  (Phyl. *Euryarchaeota*/  Class *Halobacteria*) | 7.4 | **Gen. *Haloarcula***  (Phyl. *Euryarchaeota*/  Class *Halobacteria*) | 7.7 | **Gen. *Halorubrum***  (Phyl. *Euryarchaeota*/  Class *Halobacteria*) | 4.0 |
| XKL75  (Phyl. *Euryarchaeota*/  Fam. *Halobacteriaceae*) | 3.6 | **Gen. *Haloarcula***  (Phyl. *Euryarchaeota*/  Class *Halobacteria*) | 3.8 | **Gen. *Salinibacter***  (Phyl. *Bacteroidetes*/  Class. *Rhodothermi*) | 4.8 | **MSP41**  (Phyl. *Euryarchaeota*/  Ord. *Halobacteriales*) | 7.6 | **KB1**  (Phyl. *Acetothermia*) | 4.0 |
| Gen. *Haloplanus*  (Phyl. *Euryarchaeota*/  Class *Halobacteria*) | 3.1 | **MSP41**  (Phyl. *Euryarchaeota*/  Ord. *Halobacteriales*) | 1.9 | **MSP41**  (Phyl. *Euryarchaeota*/  Ord. *Halobacteriales*) | 4.5 | **XKL75**  (Phyl. *Euryarchaeota*/  Fam. *Halobacteriaceae*) | 4.1 | **MSP41**  (Phyl. *Euryarchaeota*/  Ord. *Halobacteriales*) | 3.9 |
| MSP41  (Phyl. *Euryarchaeota*/  Ord. *Halobacteriales*) | 1.6 | **Fam. *Halobacteriaceae***  (Phyl. *Euryarchaeota*/  Class *Halobacteria*) | 1.5 | **XKL75**  (Phyl. *Euryarchaeota*/  Fam. *Halobacteriaceae*) | 4.0 | **Gen. *Planctomyces***  (Phyl. *Planctomycetes*/  Class *Planctomycetia*) | 3.6 | **Ord. *Phycisphaerales***  (Phyl. *Planctomycetes*/  Class *Phycisphaerae*) | 3.6 |
| Fam. *Halobacteriaceae*  (Phyl. *Euryarchaeota*/  Class *Halobacteria*) | 1.0 | **Gen. *Salinibacter***  (Phyl. *Bacteroidetes*/  Class. *Rhodothermi*) | 1.0 | **Fam. *Thermoanaerobacterales***  (Phyl. *Firmicutes*/  Class *Clostridia*) | 2.1 | **Fam. *Halobacteriaceae***  (Phyl. *Euryarchaeota*/  Class *Halobacteria*) | 3.6 | **XKL75**  (Phyl. *Euryarchaeota*/  Fam. *Halobacteriaceae*) | 2.9 |
|  |  |  |  | **Fam. *Halobacteriaceae***  (Phyl. *Euryarchaeota*/  Class *Halobacteria*) | 1.8 | **Fam. *Halobacteriaceae***  (Phyl. *Euryarchaeota*/  Class *Halobacteria*) | 3.0 | **Ord. *Acetothermales***  (Phyl. *Acetothermia*) | 2.4 |
|  |  |  |  | **Fam. *Thiohalorhabdaceae***  (Phyl. *Proteobacteria*/  Class *Gammaproteobacteria*) | 1.3 | **Fam *Pirellulaceae***  (Phyl. *Planctomycetes*/  Class *Planctomycetia*) | 2.4 | **Fam. *Thiohalorhabdaceae***  (Phyl. *Proteobacteria*/  Class *Gammaproteobacteria*) | 2.2 |
|  |  |  |  | **Fam. *Halobacteriaceae***  (Phyl. *Euryarchaeota*/  Class *Halobacteria*) | 1.1 | **Fam. *Thiohalorhabdaceae***  (Phyl. *Proteobacteria*/  Class *Gammaproteobacteria*) | 1.6 | **Fam. *Halobacteriaceae***  (Phyl. *Euryarchaeota*/  Class *Halobacteria*) | 1.7 |
|  |  |  |  | **Ord. *Phycisphaerales***  (Phyl. *Planctomycetes*/  Class *Phycisphaerae*) | 1.1 | **Ord. *Phycisphaerales***  (Phyl. *Planctomycetes*/  Class *Phycisphaerae*) | 1.5 | **Gen. *Planctomyces***  (Phyl. *Planctomycetes*/  Class *Planctomycetia*) | 1.4 |
|  |  |  |  |  |  | **Ord. *Acetothermales***  (Phyl. *Acetothermia*) | 1.4 | **Fam. *Thermoanaerobacterales***  (Phyl. *Firmicutes*/  Class *Clostridia*) | 1.2 |
|  |  |  |  |  |  | **Gen. *Halomicrobium***  (Phyl. *Euryarchaeota*/  Class *Halobacteria*) | 1.3 | **Fam. *Halanaerobiaceae***  (Phyl. Firmicutes/  Class Clostridia) | 1.2 |
|  |  |  |  |  |  | **Fam. *Thermoanaerobacterales***  (Phyl. *Firmicutes*/  Class *Clostridia*) | 1.0 | **Phyl. *Planctomycetes*** | 1.1 |
|  |  |  |  |  |  |  |  | **Fam *Pirellulaceae***  (Phyl. *Planctomycetes*/  Class *Planctomycetia*) | 1.0 |
